# Supplementary material for: Mechanisms of MCM2–7 helicase activation and initial DNA melting at near base-pair resolution
Source: Nat Commun. 2026 Jul 23;17:8984. doi: 10.1038/s41467-026-75695-1 (PMC13507265; doi:10.1038/s41467-026-75695-1)
Supplement: Supplementary file 1 [file 41467_2026_75695_MOESM1_ESM.pdf]

SUPPLEMENTARY INFORMATION

Mechanisms of MCM2-7 helicase activation and initial DNA melting at near base-pair resolution

Christopher Weekes, Lia Willerding, Sanjay P. Khadayate, Korbinian Liebl, Audrey Mossler, Alex Montoya, Vanessa Rauthe, Mohammad M. Karimi, Martin Zacharias, Helle D. Ulrich, Christian Speck, and L. Maximilian Reuter

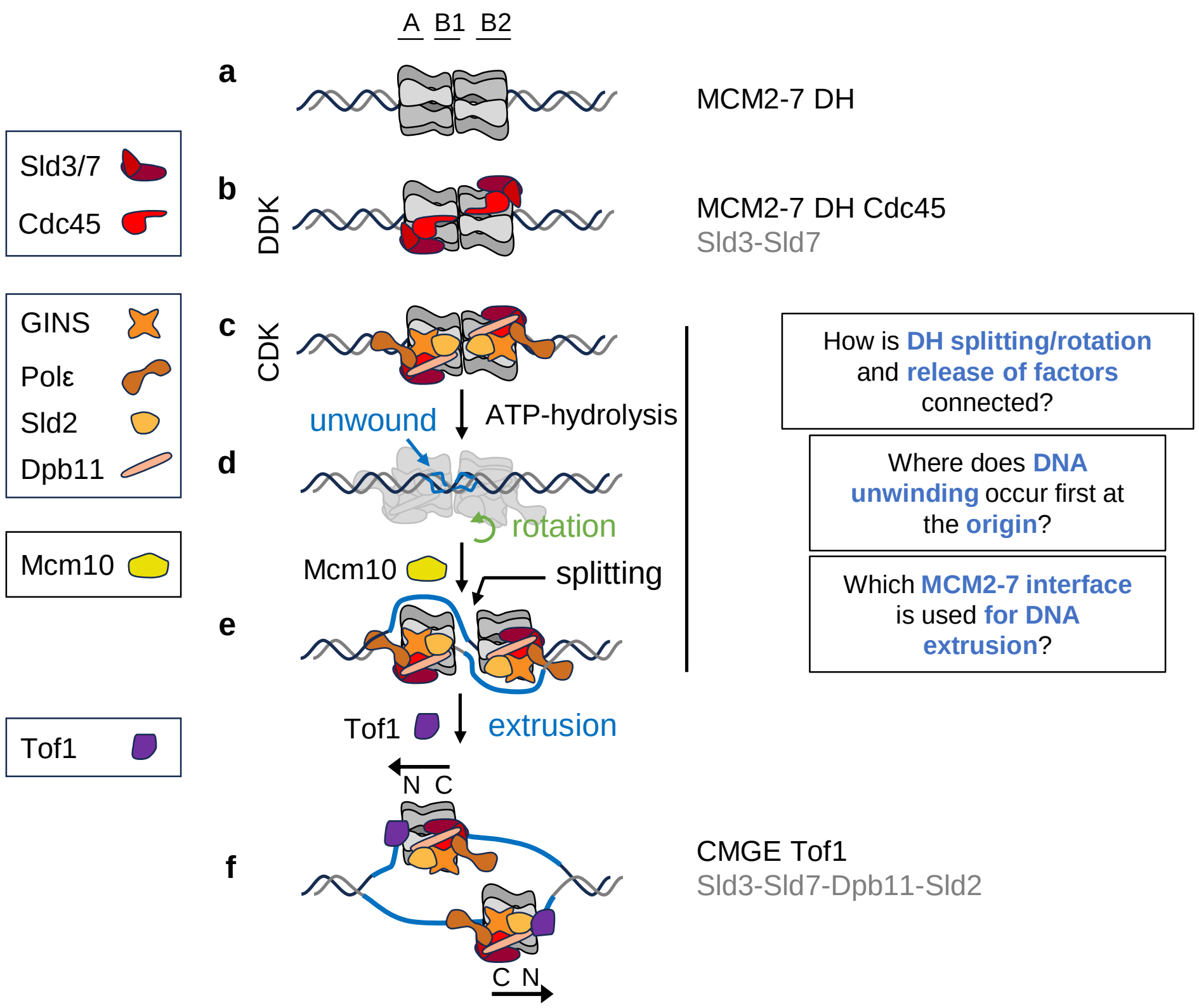

Supplementary Figure 1: MCM2-7 helicase activation pathway in budding yeast.

(**a**) MCM2-7 double-hexamer (DH). (**b**) DDK-activated MCM2-7 DH in complex with Sld3-Sld7. (**c**) CDK activity promotes recruitment of helicase activation factors GINS, Polε, Sld2, and Dpb11. (**d**) Sld2-GINS-Dpb11-Polε-induced MCM2-7 ATPase-dependent activity results in the splitting of the DH and a rotation of the two CMGE complexes against each other. (**e**) Mcm10 recruitment promotes DNA extrusion and complete separation of the two hexamers. (**f**) Extended unwinding promotes the recruitment of Tof1, part of the replication fork protection complex. Loading factors that do not form part of the replication fork are shown in grey.

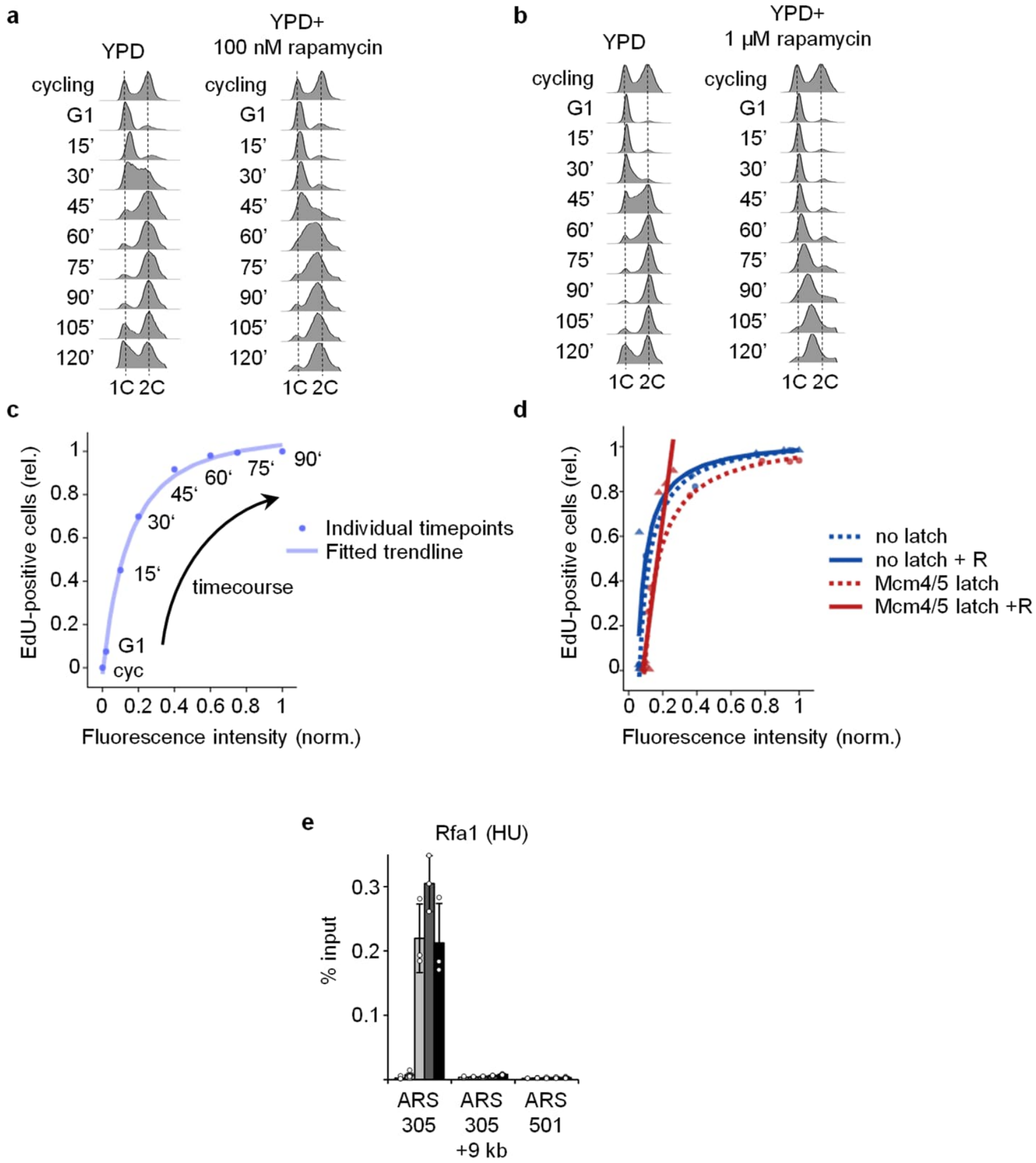

Supplementary Figure 2: Analysis of the Mcm4/5 latch linkage.

(**a**) Representative flow cytometry profiles of the inter-hexamer latch strain with and without rapamycin treatment. Cells were released into S-phase in the presence or absence of rapamycin after alpha-factor synchronisation. (**b**) As for (a), but with the addition of 1 µM rapamycin, showing improved rapamycin-dependent initiation delay. (**c**) Schematic figure of the composite plots in (d) and Fig. 3. Cells incorporating EdU were arrested and released in G1 and released into S-phase synchronously. The percentage of cells positive for EdU incorporation (y-axis) and the overall normalised median fluorescence intensity of the EdU staining (x-axis) are displayed over a time course of 90 minutes.(**d**) Composite plot of an EdU incorporation time course of the Mcm4/5 inter-hexamer linkage (red) and a correspodnign wt (blue) in the absence (dotted lines) and presence of rapamycin (+R, 1 µM, solid lines). α–factor arrested cells were treated with rapamycin before release into S-phase for 90 minutes, and samples were taken every 15 min. Strains are colour-coded, and individual dots represent the relative percentage of EdU-positive cells and the geometric mean of EdU fluorescence intensity of 40,000 cells at a given time point (cyc, G1-90 min). Related to Fig. 1e and f. (**e**) Rfa1 recruitment to *ARS305*, 9 kb distal to *ARS305* (*ARS305*+9kb), and *ARS501*. An Rfa1-5xFlag strain was harvested at the indicated timepoints after α-factor arrest and release at 25°C for the indicated amounts of time in full media complemented with 200 mM Hydroxyurea. qPCR data are the average and standard deviation derived from three biological replicates. Source data is provided as a Source Data file.

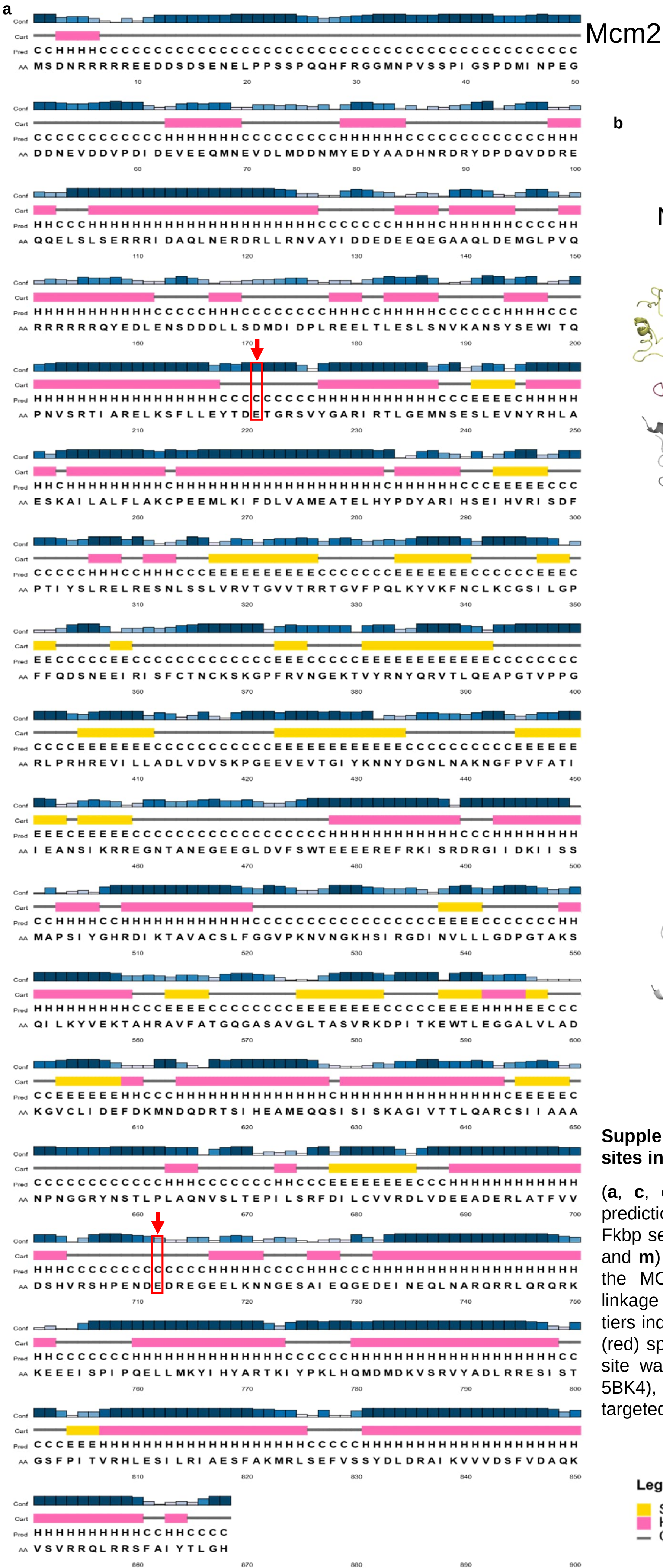

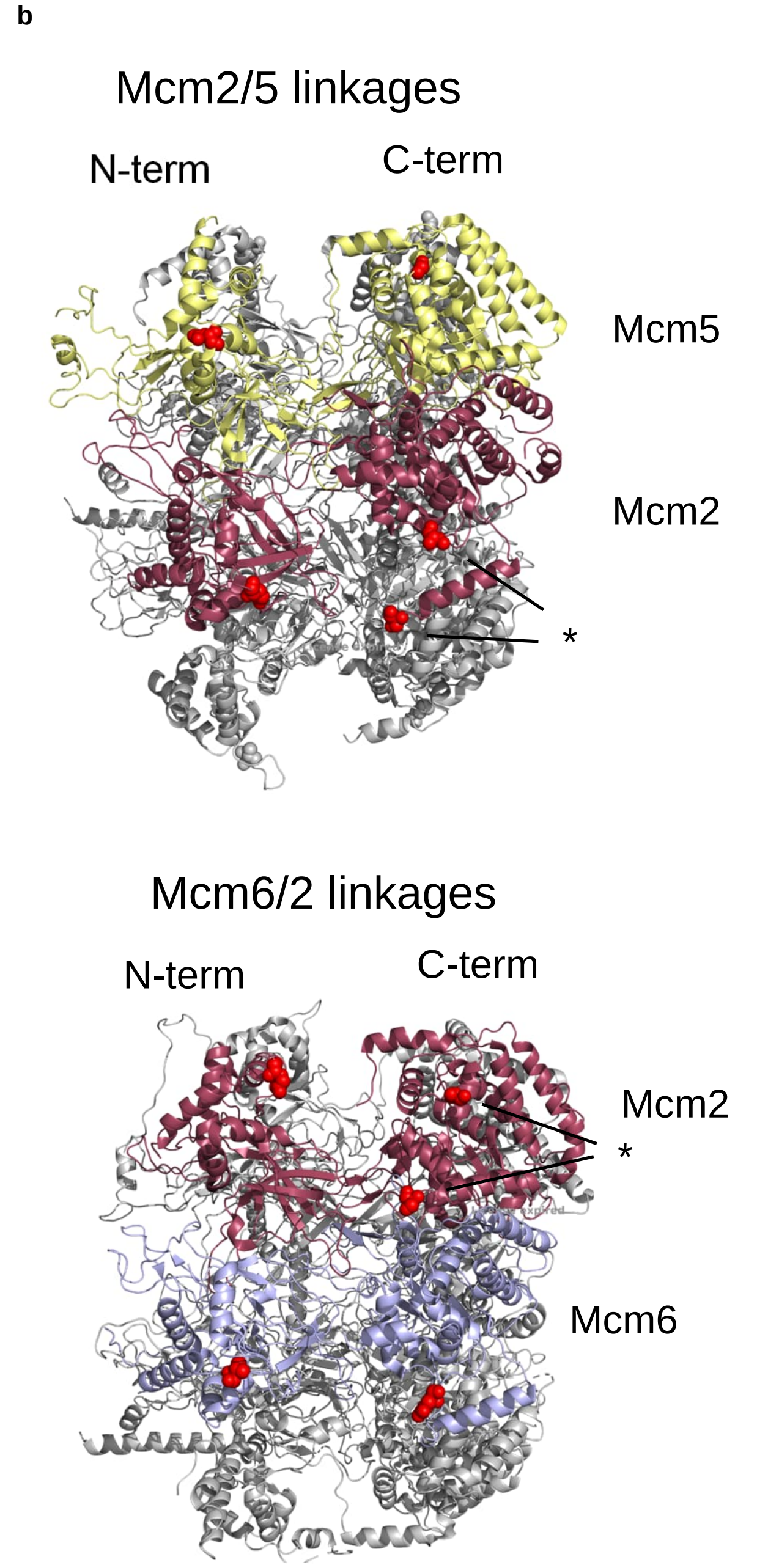

Supplementary Figure 3: FRB-/ FKBP-integration sites in the Mcm subunits of MCM2-7.

(**a**, **c**, **e**, **g**, **i**, and **k**) PSIPRED secondary structure predictions with highlighted integration sites for Frb and Fkbp sequences (red boxes with arrows). (**b**, **d**, **f**, **h**, **j**, **l**, and **m**) Structural representation of the insertion sites in the MCM2-7 complex. MCM2-7 subunits involved in linkage formation are colour-coded, N- and C-terminal tiers indicated, and insertion sites are shown as coloured (red) spheres. An asterisk (*) indicates that the insertion site was not resolved in the Cryo-EM structure (PDB: 5BK4), so the first adjacent amino acids bordering the targeted, unresolved, and flexible loop are highlighted.

Legend:

Strand
Helix
Coil

Conf: - + Confidence of prediction
Cart: 3-state assignment cartoon
Pred: 3-state prediction
AA: Target Sequence

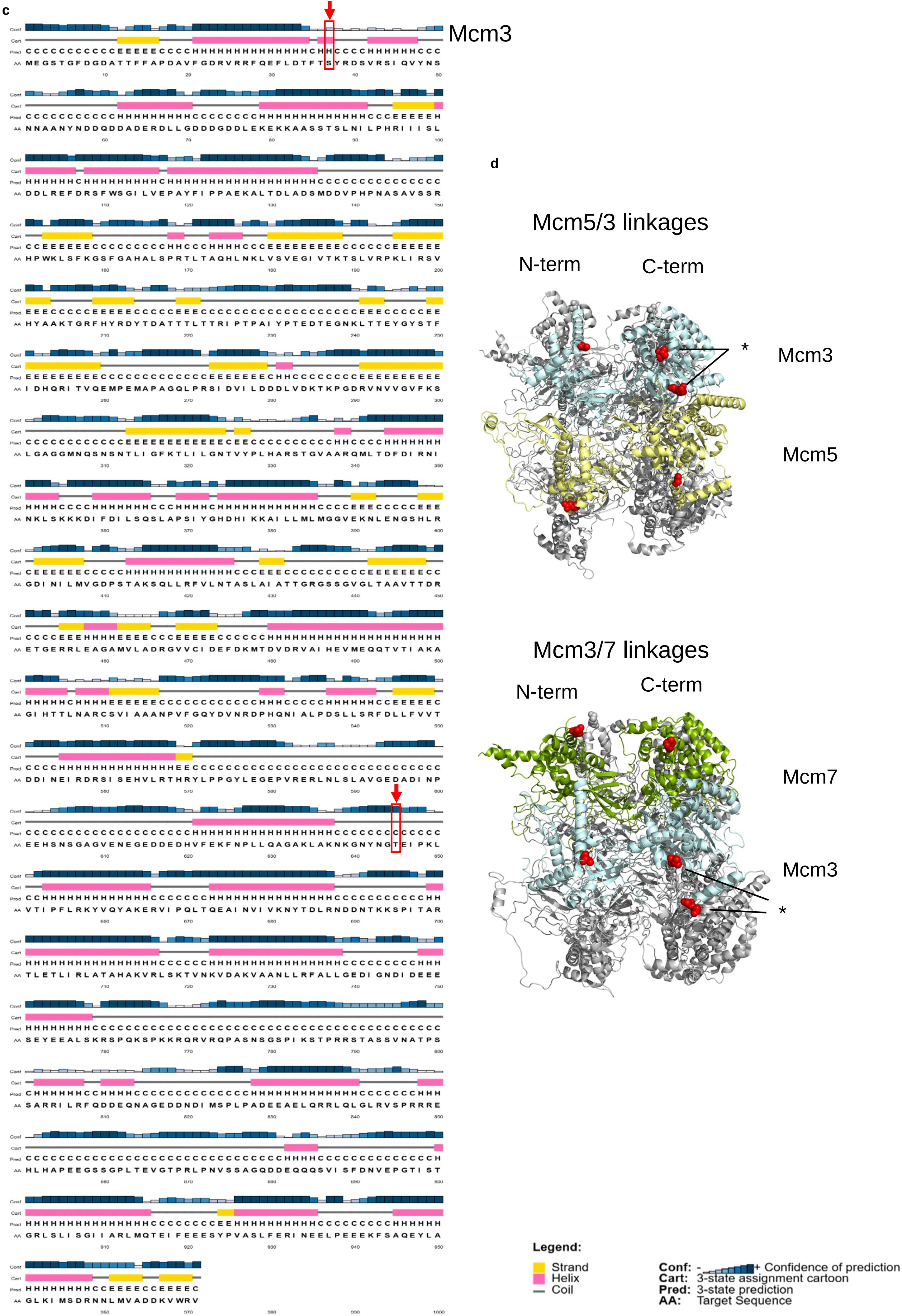

c
Mcm3
d
Mcm5/3 linkages
N-term
C-term
Mcm3
Mcm5
*
Mcm3/7 linkages
N-term
C-term
Mcm7
Mcm3
*
Legend:
Strand
Helix
Coil
Conf: Confidence of prediction
Cart: 3-state assignment cartoon
Pred: 3-state prediction
AA: Target Sequence

e

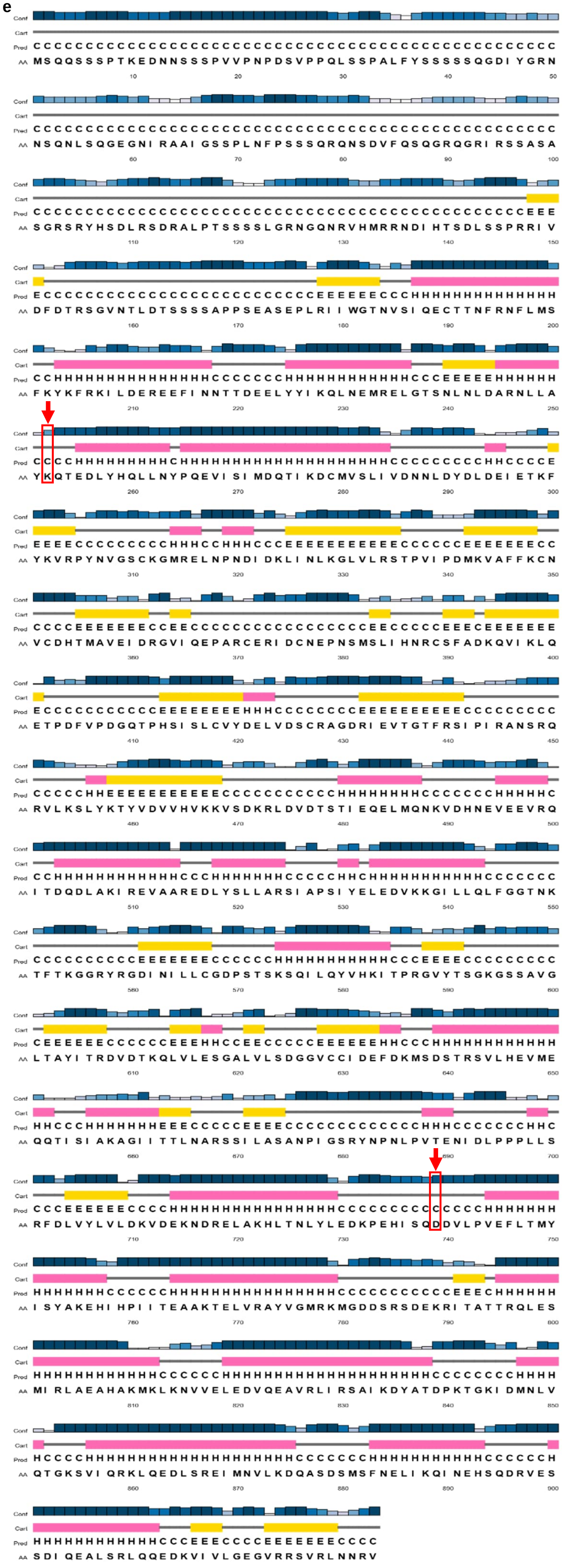

Mcm4

f

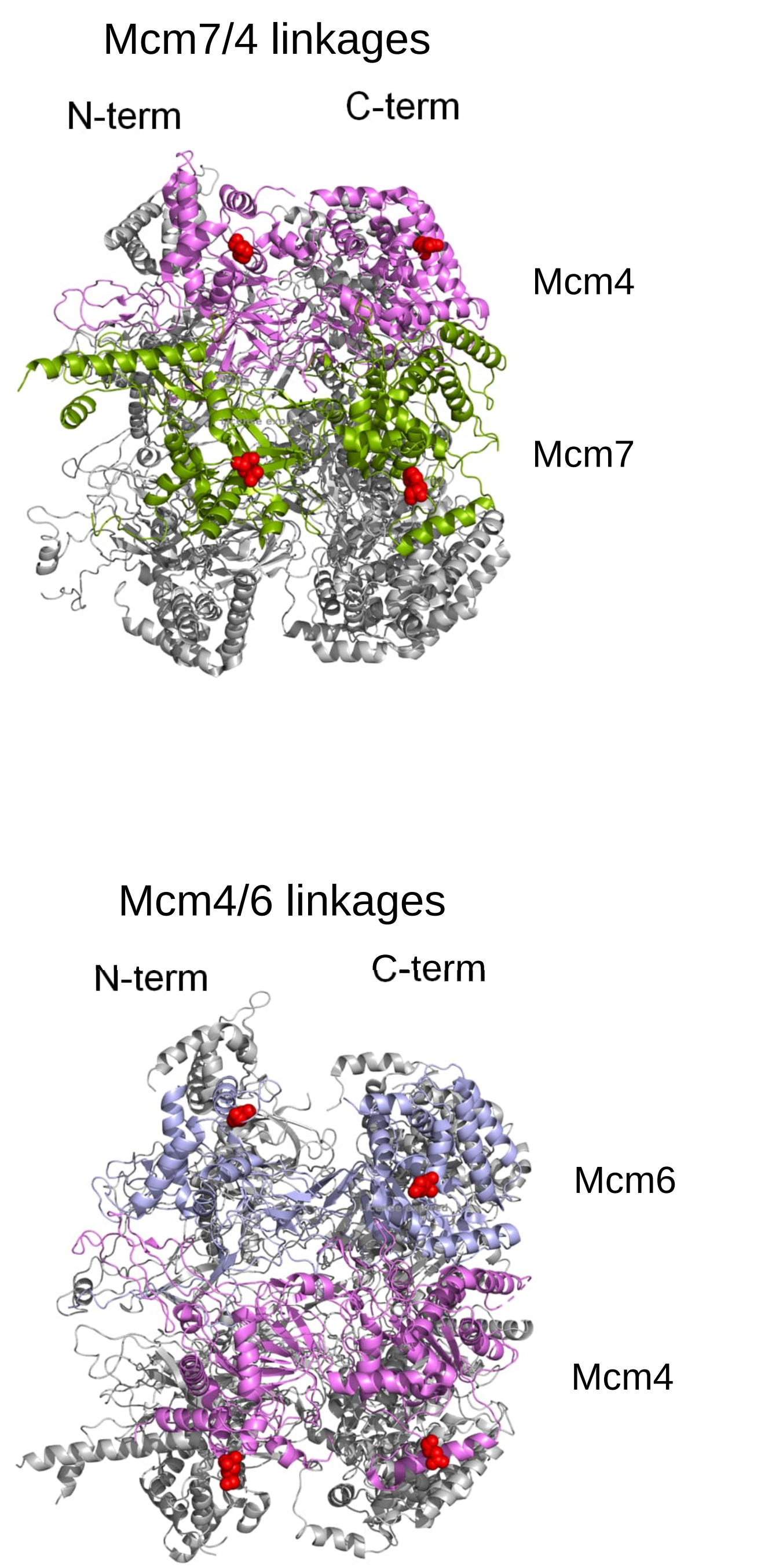

Legend:

Strand
Helix
Coil

Conf: - + Confidence of prediction
Cart: 3-state assignment cartoon
Pred: 3-state prediction
AA: Target Sequence

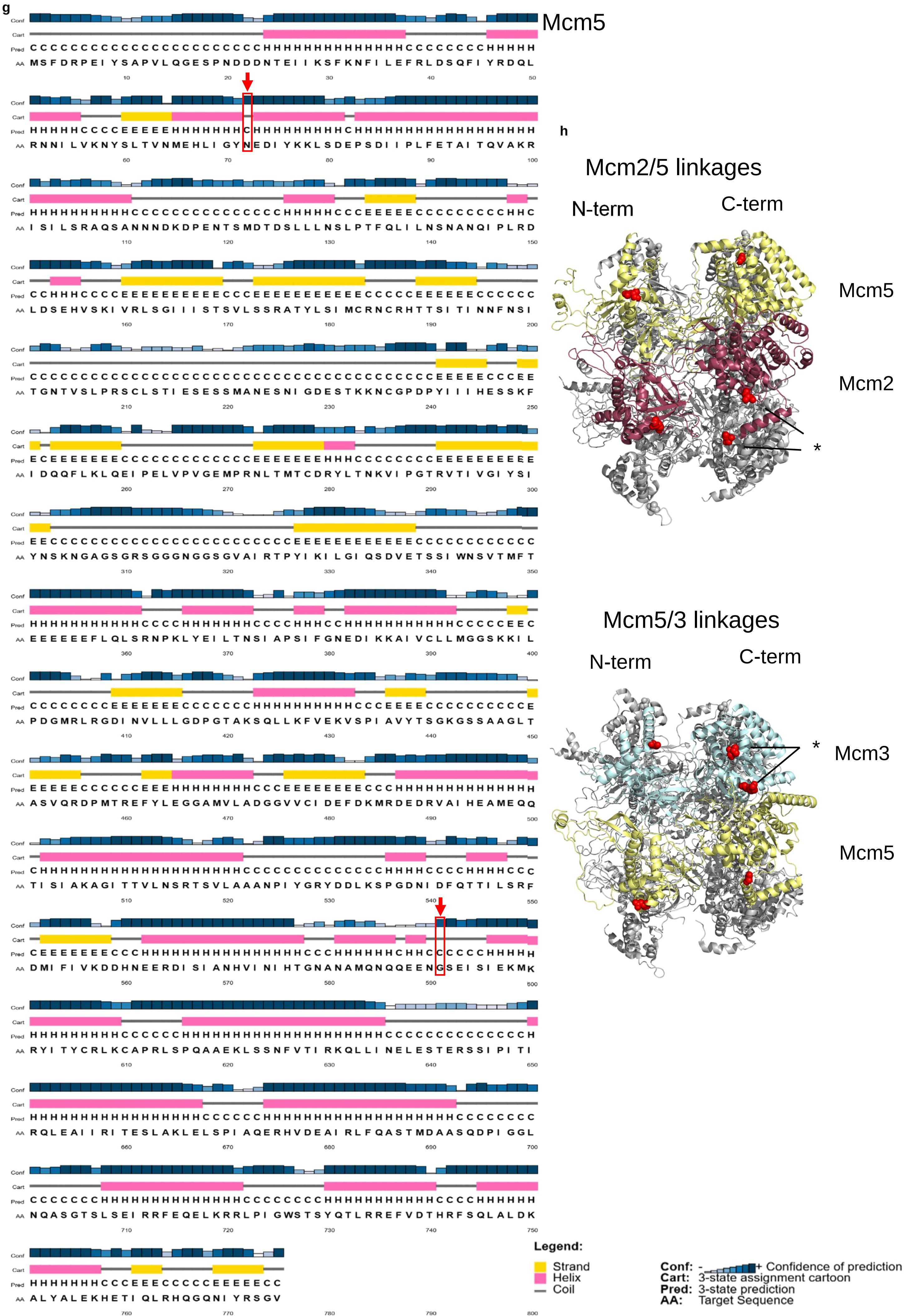

g
Mcm5
Conf
Cart
Pred
AA
CCCCCCCCCCCCCCCCCCCCCCCHHHHHHHHHHHHHHCCCCCCCCHHHHH
MSFDRPEIYSAPVLQGESPNDDDNTEIIKSFKNFILEFRLDSQFIYRDQL
10 20 30 40 50
HHHHHCCCCEEEEEEHHHHHHHCHHHHHHHHHCHHHHHHHHHHHHHHHHH
RNNILVKNYSLTVNMEHLIGYNEDIYKKLSDEPSDIIPLFETAITQVAKR
60 70 80 90 100
HHHHHHHHHHCCCCCCCCCCCCCCCHHHHHCCCEEEEEECCCCCCCCCHHC
ISILSRAQSANNNDKDPENTSMDTDSLLLNSLPTFQLILNSNANQIPLRD
110 120 130 140 150
CCHHHCCCCEEEEEEEEEEECCCEEEEEEEEEEEECCCCCEEEEEECCCCCC
LDSEHVSKIVRLSGIIISTSVLSSRATYLSIMCRNCRHTTSITINNFNSI
160 170 180 190 200
CCCCCCCCCCCCCCCCCCCCCCCCCCCCCCCCCCCCCCCEEEEECCCEE
TGNTVSLPRSCLSTIESESSMANESNIGDESTKKNCGPDPYIIIHESSKF
210 220 230 240 250
ECEEEEEEEECCCCCCCCCCCCCEEEEEEEHHHCCCCCCCCEEEEEEEEEE
IDQQFLKLQEIPELVPVGEMPRNLTMTCDRYLTNKVIPGTRVTIVGIYSI
260 270 280 290 300
EECCCCCCCCCCCCCCCCCCCCCCCCCEEEEEEEEEEEECCCCCCCCCCC
YNSKNGAGSGRSGGGNGGSGVAIRTPYIKILGIQSDVETSSIWNSVTMFT
310 320 330 340 350
HHHHHHHHHHHCCCCHHHHHHHCCCCHHHCCHHHHHHHHHHHCCCCCEEC
EEEEEEFLQLSRNPKLYEILTNSIAPSIFGNEDIKKAIVCLLMGGSKKIL
360 370 380 390 400
CCCCCCCCEEEEEEECCCCCCCHHHHHHHHHHCCCEEEECCCCCCCCCCE
PDGMRLRGDINVLLLGDPGTAKSQLLKFVEKVSPIAVYTSGKGSSAAGLT
410 420 430 440 450
EEEEECCCCCCEEEHHHHHHHHCCCEEEEEEEEECCHHHHHHHHHHHHHH
ASVQRDPMTREFYLEGGAMVLADGGVVCIDEFDKMRDEDRVAIHEAMEQQ
460 470 480 490 500
CHHHHHHHHHHHHHHHHHHHHCCCCCCCCCCCCCCHHHHCCCHHHHHCCC
TISIAKAGITTVLNSRTSVLAAANPIYGRYDDLKSPGDNIDFQTTILSRF
510 520 530 540 550
CEEEEEEECCHHHHHHHHHHHHHHHHHCCHHHHHHHCHHCCCCCHHHHH
DMIFIVKDDHNEERDISIANHVINIHTGNANAMQNQQEENGSEISIEKMK
560 570 580 590 600
HHHHHHHHHCCCCCCHHHHHHHHHHHHHHHHHHHHCCCCCCCCCCCCCCH
RYITYCRLKCAPRLSPQAAEKLSSNFVTIRKQLLINELESTERSSIPITI
610 620 630 640 650
HHHHHHHHHHHHHHHHHCCCCCCHHHHHHHHHHHHHHHHHHHCCCCCCC
RQLEAIIRITESLAKLELSPIAQERHVDEAIRLFQASTMDAASQDPIGGL
660 670 680 690 700
CCCCCCCHHHHHHHHHHHHHHHHCCCCCCCCHHHHHHHHHHHHCCCCHHHHHH
NQASGTSLSEIRRFEQELKRRLPIGWSTSYQTLRREFVDTHRFSQLALDK
710 720 730 740 750
HHHHHHHCCCEEEECCCCCEEEEEECC
ALYALEKHETIQLRHQGQNIYRSGV
760 770 780 790 800
Legend:
Strand
Helix
Coil
Conf: Confidence of prediction
Cart: 3-state assignment cartoon
Pred: 3-state prediction
AA: Target Sequence
h
Mcm2/5 linkages
N-term
C-term
Mcm5
Mcm2
*
Mcm5/3 linkages
N-term
C-term
*
Mcm3
Mcm5

i

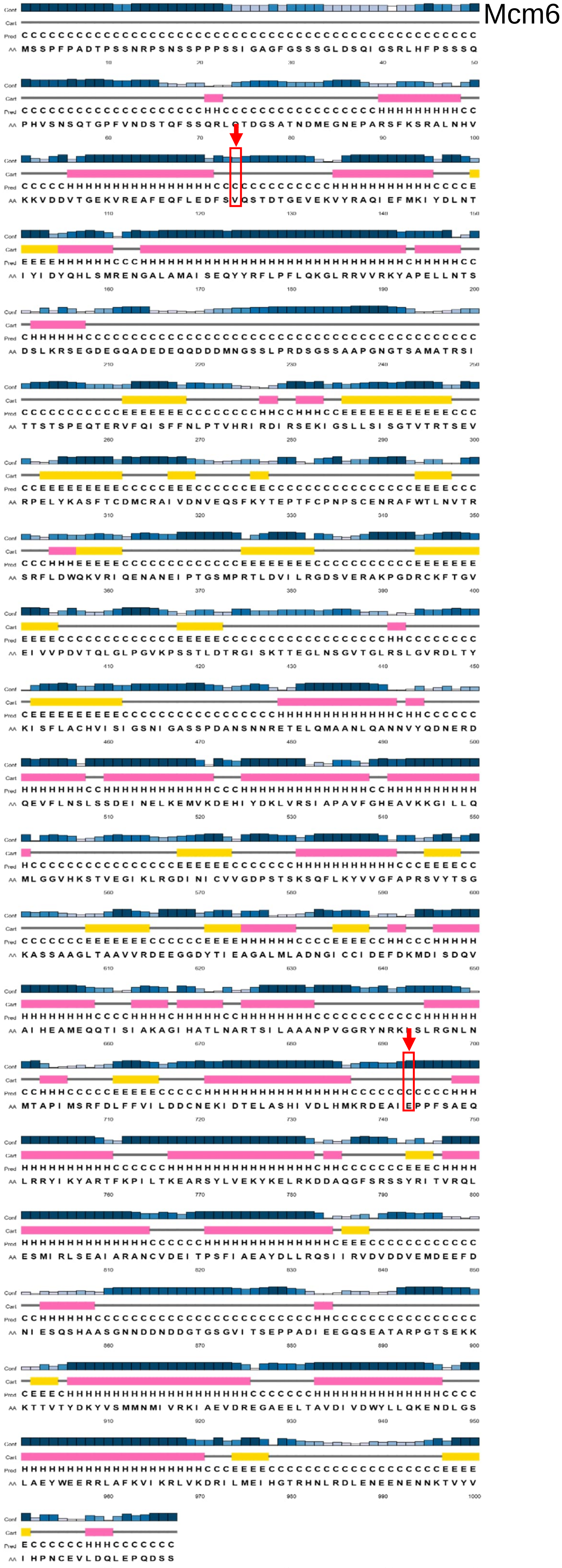
Mcm6
Conf
Cart
Pred
AA
Legend:
Strand
Helix
Coil
Conf: - + Confidence of prediction
Cart: 3-state assignment cartoon
Pred: 3-state prediction
AA: Target Sequence

j

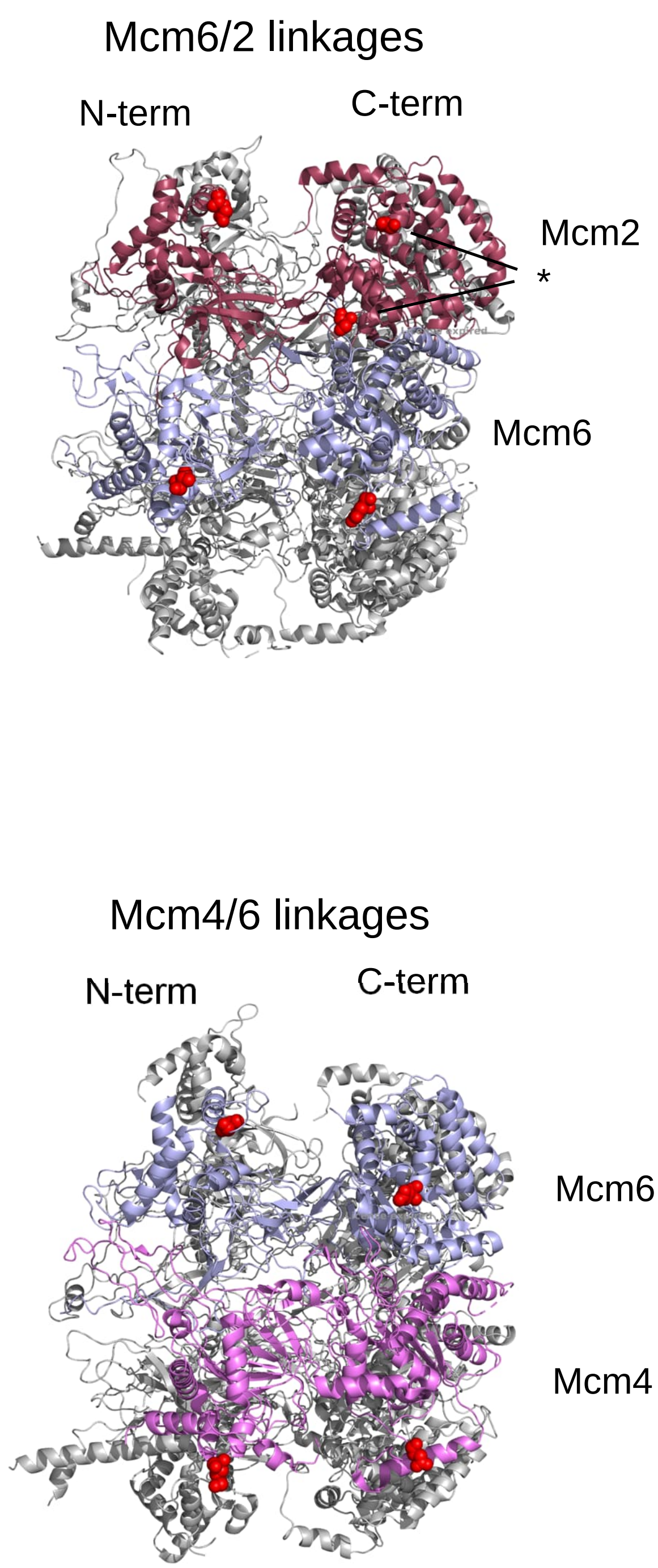
Mcm6/2 linkages
N-term
C-term
Mcm2
*
Mcm6
Mcm4/6 linkages
N-term
C-term
Mcm6
Mcm4

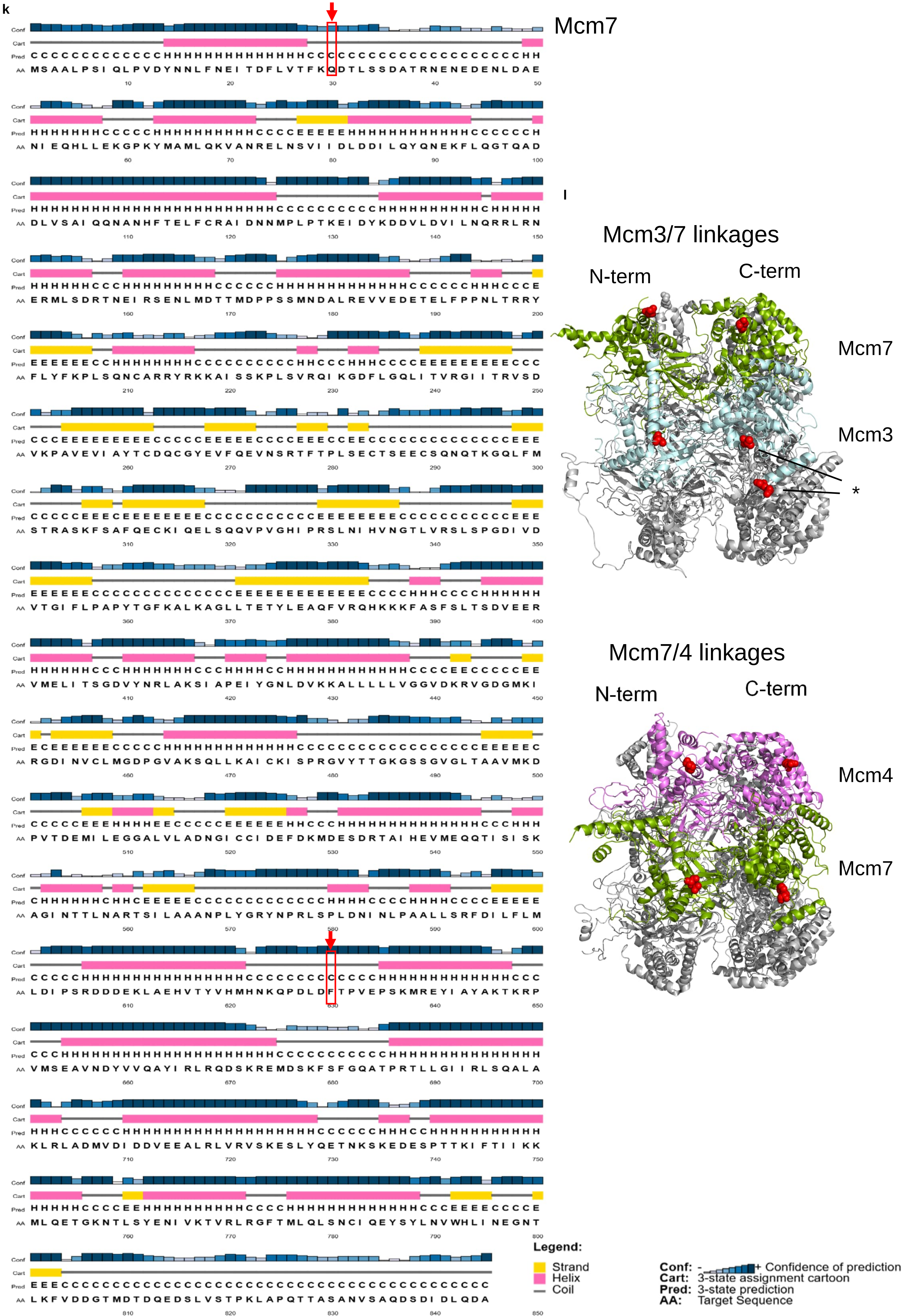
k
Mcm7
Conf
Cart
Pred
AA
l
Mcm3/7 linkages
N-term
C-term
Mcm7
Mcm3
*
Mcm7/4 linkages
N-term
C-term
Mcm4
Mcm7
Legend:
Strand
Helix
Coil
Conf: Confidence of prediction
Cart: 3-state assignment cartoon
Pred: 3-state prediction
AA: Target Sequence

m

Mcm4/5 linkage (latch)

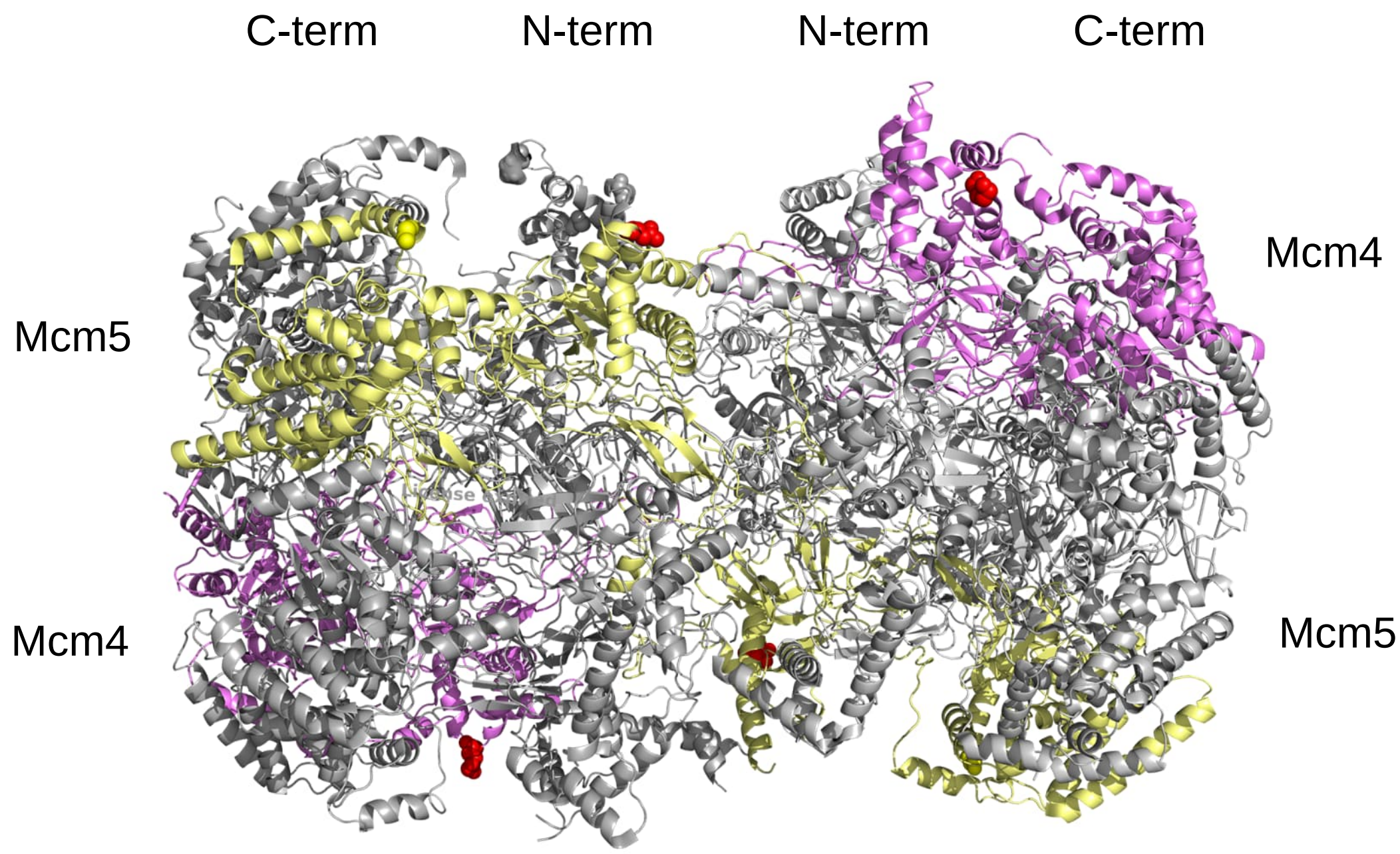

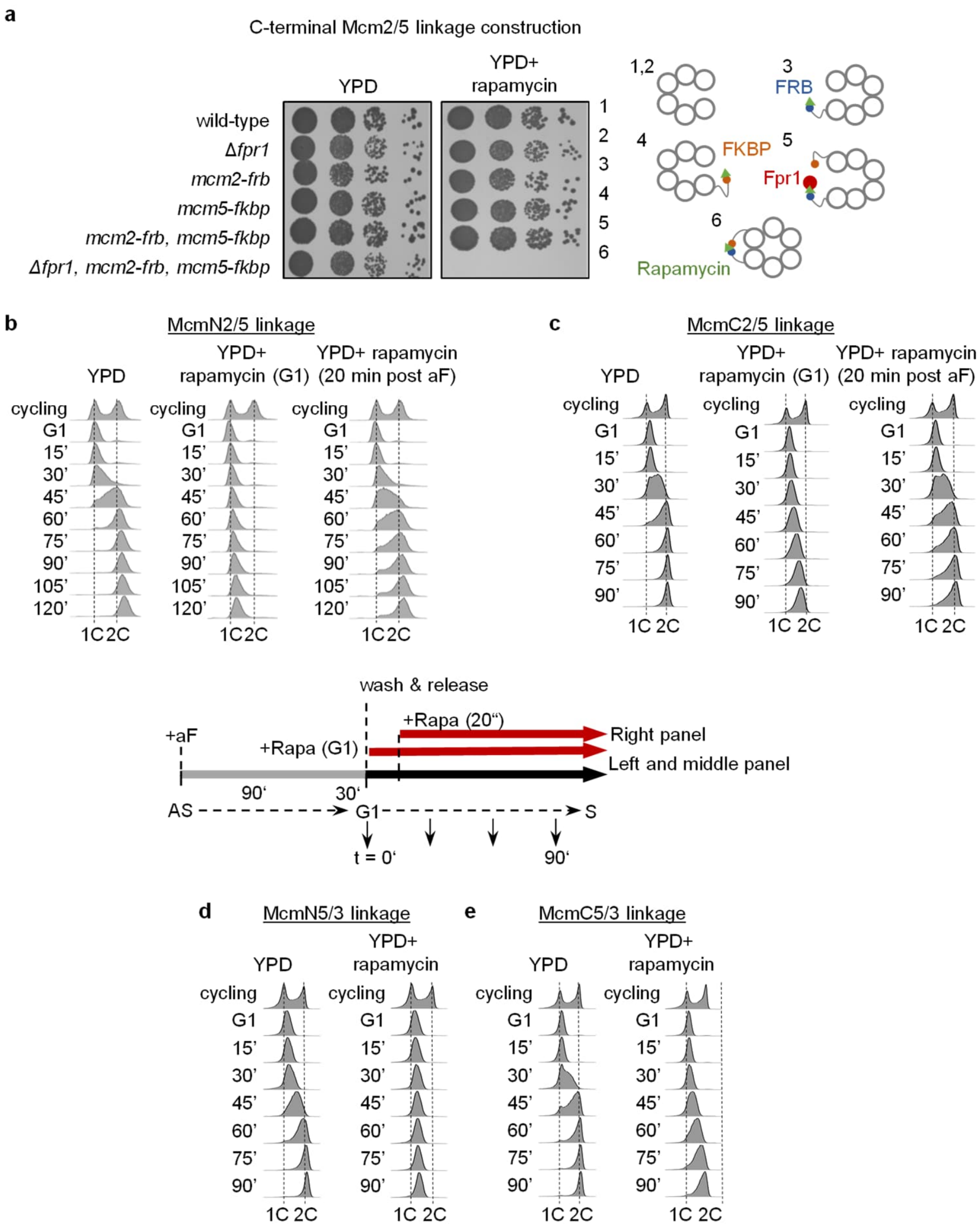

Supplementary Figure 4: The Mcm2/5 linkage constructs result in specific cell cycle arrests.

(**a**) The rapamycin-dependent lethal phenotype of the closed Mcm2/5 linkage relies on a complete linkage construct. Dot spot assay of cells from various construction stages of the C-terminal Mcm2/5 linkage mutant on full media (YPD) or YPD-rapamycin (100 nM). Only the complete mutant strain harbouring all necessary genomic integrations is sensitive to rapamycin treatment. FRB/FKBP domains with flexible linkers (blue and red dots with lines) and Rapamycin (green triangle) are shown next to the schematic MCM2-7 subunits. (**b** and **c**) The cell cycle arrest of the Mcm2/5 linkages is dependent on linkage closure before S-phase commitment. Representative flow cytometry profiles of a strain harbouring an (**b**) N-terminal Mcm2/5 linkage or (**c**) C-terminal Mcm2/5 linkage in the absence (left panel) and presence of rapamycin (100 nM, middle and right panels). Cycling cells were arrested for 2 hrs using α–factor and treated with rapamycin either at the end of the G1-phase arrest (middle panel) or 20 minutes after release from the α–factor arrest (right panel). (**d** and **e**) Representative flow cytometry profiles of strains harbouring an (**d**) N-terminal or (**e**) C-terminal Mcm5/3 linkage in the absence (left panel) and presence of rapamycin (100 nM, right panel). Cycling cells were arrested for 2 hrs using α-factor and treated with rapamycin before release into S-phase.

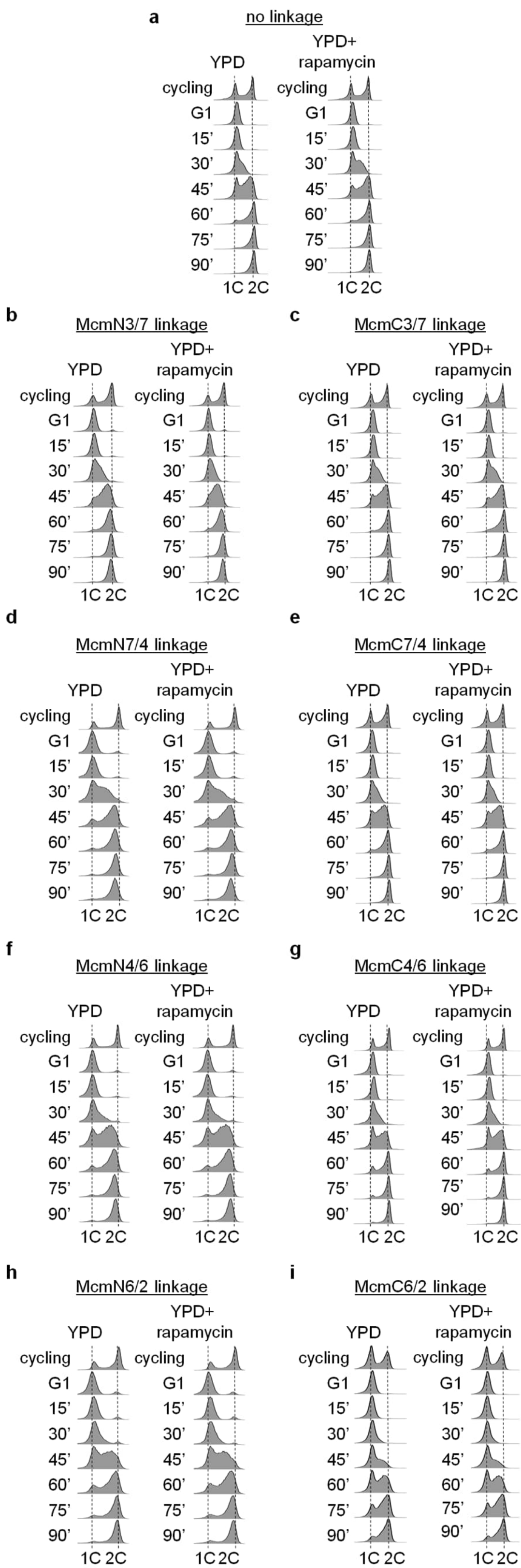

Supplementary Figure 5: Flow cytometry profiles of the remaining N- and C- terminal Mcm2-7 linkages.

Representative flow cytometry profiles of strains without an (**a**) FRB/FKBP linkage, (**b**, **d**, **f**, and **h**) N-terminal, or (**c**, **e**, **g**, and **i**) C-terminal Mcm linkages in the absence and presence of rapamycin (100 nM). Cycling cells were arrested for 2 hrs and treated with rapamycin before release into S-phase for 90 minutes. (**b** and **c**) Cytometry profiles for an (**b**) N-terminal or (**c**) C-terminal Mcm3/7 linkage strain. (**d** and **e**) Cytometry profiles for an (**d**) N-terminal or (**e**) C-terminal Mcm7/4 linkage strain. (**f** and **g**) Cytometry profiles for an (**f**) N-terminal or (**g**) C-terminal Mcm4/6 linkage strain. (**h** and **i**) Cytometry profiles for an (**h**) N-terminal or (**i**) C-terminal Mcm6/2 linkage strain.

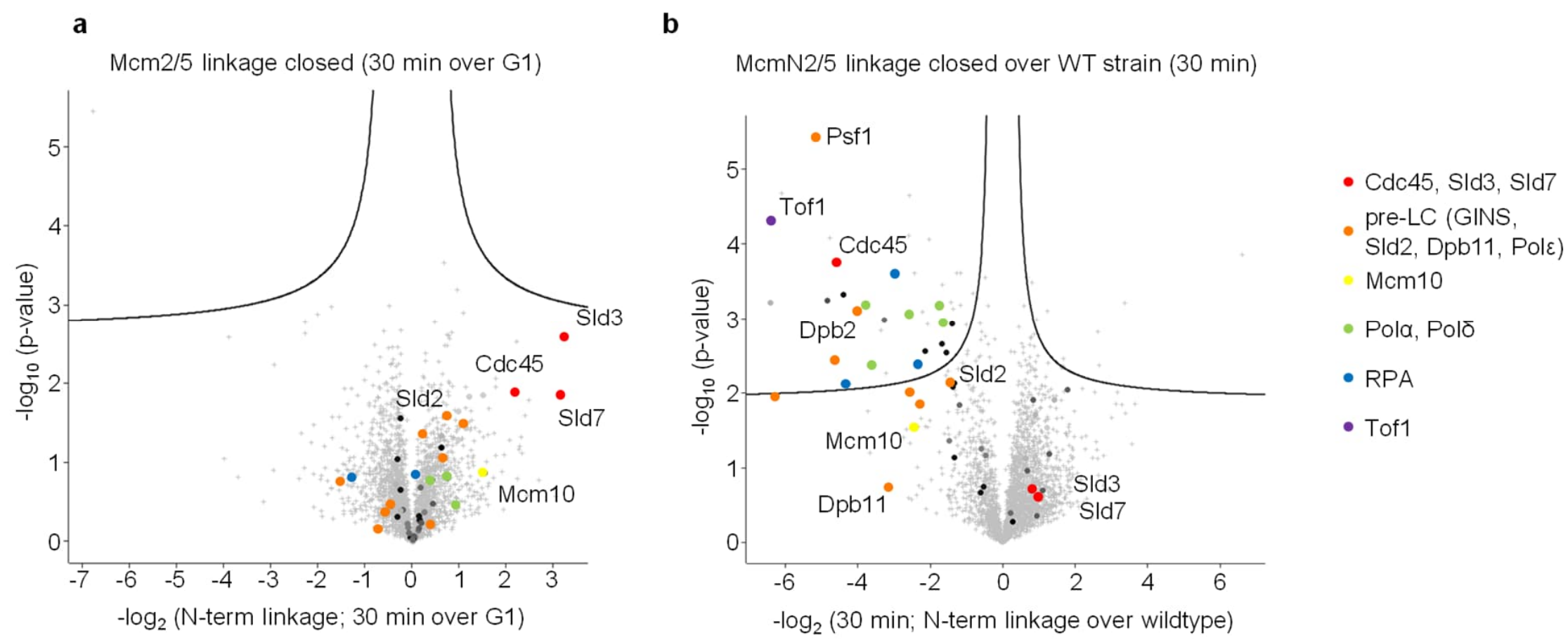

Supplementary Figure 6: The N-terminal Mcm2/5 linkage blocks CMG remodelling.

(**a** and **b**) The Mcm2/5 N-terminal linkage construct arrests CMG assembly at an early stage. (**a**) Volcano plot summarising replication factor enrichments of Mcm4-containing complexes from cells released into S-phase for 30 min versus G1-arrested cells in the context of the closed Mcm2/5 linkage. Important replication initiation factors are colour-coded: Cdc45, Sld3/7 (red), pre-LC components (orange), Mcm10 (yellow), Pol1 and 3 (green), RPA (blue) and Tof1 (purple). (**b**) The McmN2/5 linkage impedes MCM2-7 helicase maturation during early activation. Volcano plot summarising replication factor enrichments of Mcm4-containing complexes from closed McmN2/5 linkage cells over wild-type cells released into S-phase for 30 min. Replication initiation factors are colour-coded as in (a). Source data is provided as a Source Data file.

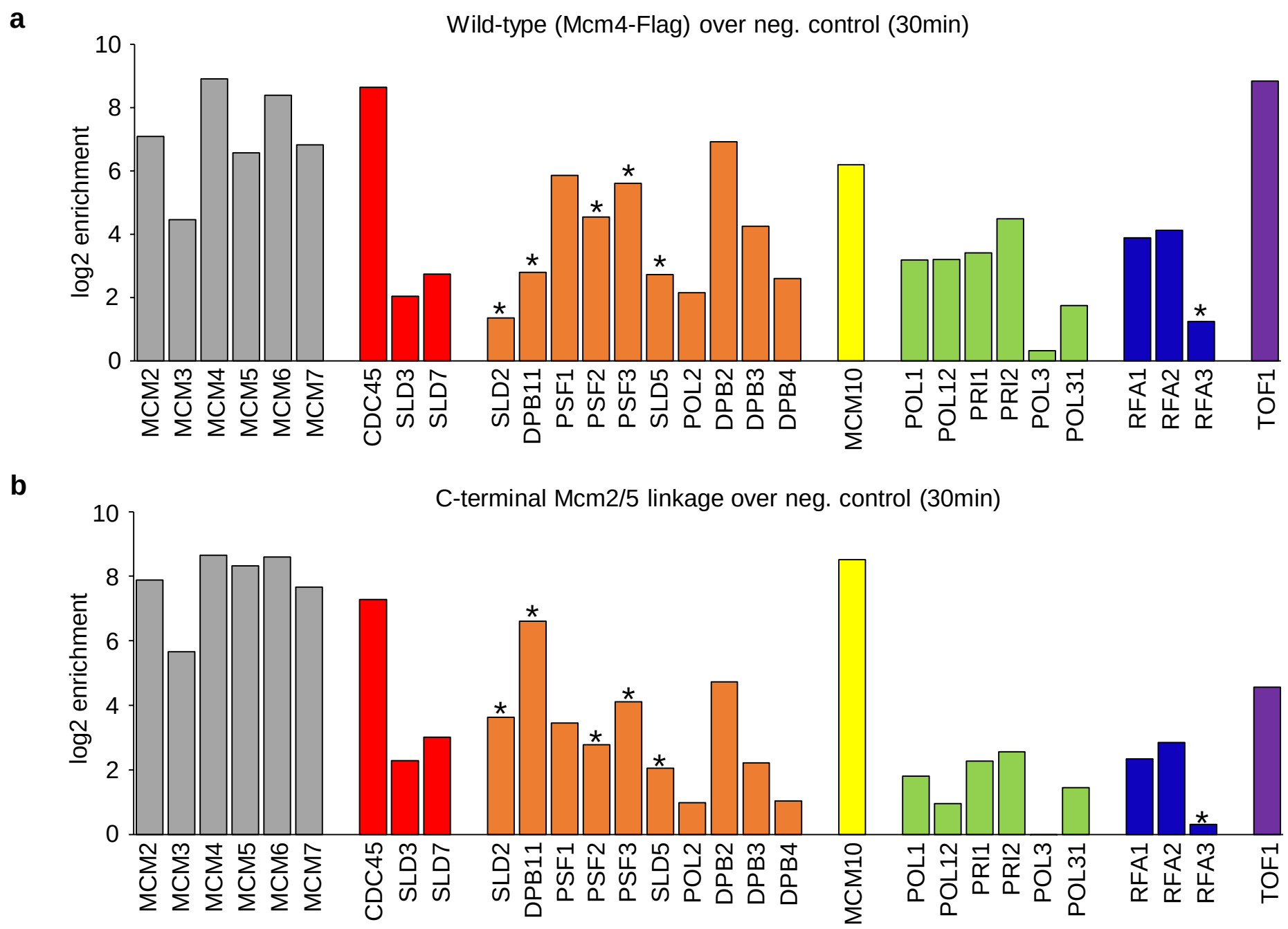

Supplementary Figure 7: Protein composition of the WT and arrested McmC2/5 linkage complex.

MCM2-7 complexes are enriched for proteins involved in replication activation and fork assembly. (**a**) Mcm4 ChIP-MS enrichment of core replisome factors in the wild-type strain, missing the linkage in G1-phase. Enrichment values are given as the log_2 of the mean LFQ enrichment of subject proteins in the Mcm4-5xFLAG strain (IP) over a negative control (NC – no Flag tag present) strain from four independent replicates. Asterisks mark proteins that lacked intensity measurements in the NC. (**b**) As in (**a**), showing Mcm4 ChIP-MS enrichment of core replisome factors in the Mcm2/5 C-terminal linkage strain released into S-phase for 30 min over a negative control (NC – no Flag tag present) strain. (**b**) is a copy of Fig. 4b, allowing for easier comparison of the data. Source data is provided as a Source Data file.

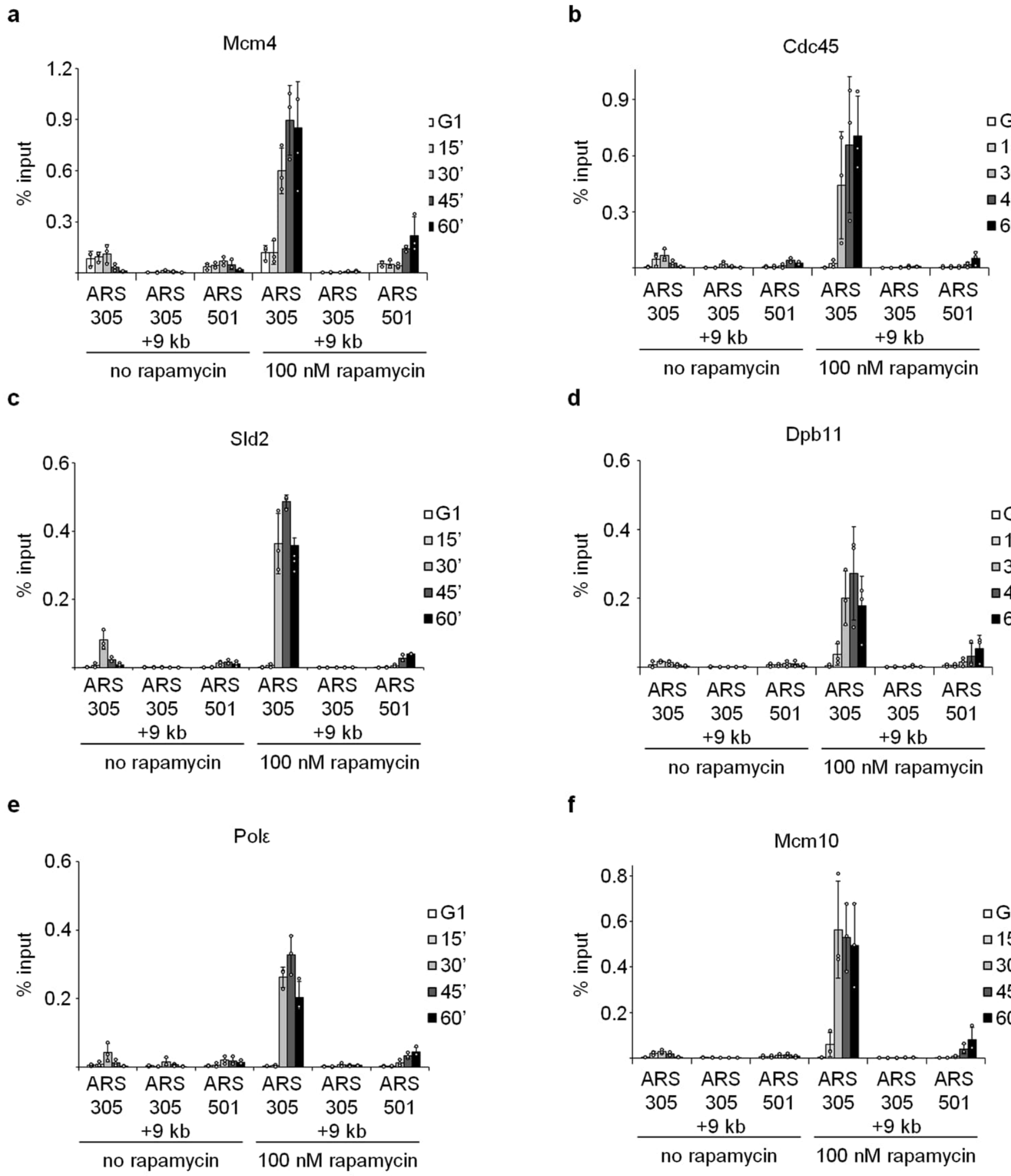

Supplementary Figure 8: Recruitment of replication factors to origins in the presence of C-terminal Mcm2/5 linkage construct.

ChIP-qPCR profiles of (**a**) Mcm4, (**b**) Cdc45, (**c**) Sld2, (**d**) Dpb11, (**e**) Polε, and (**f**) Mcm10 on *ARS305*, 9 kb distal to *ARS305* (*ARS305*+9 kb) and *ARS501*. Tagged Mcm2/5 C-terminal linkage strains were treated with or without rapamycin (100 nM) and harvested at the indicated timepoints after α-factor arrest and release at 25 °C. qPCR data are the average and standard deviation derived from three biological replicates. Source data is provided as a Source Data file.

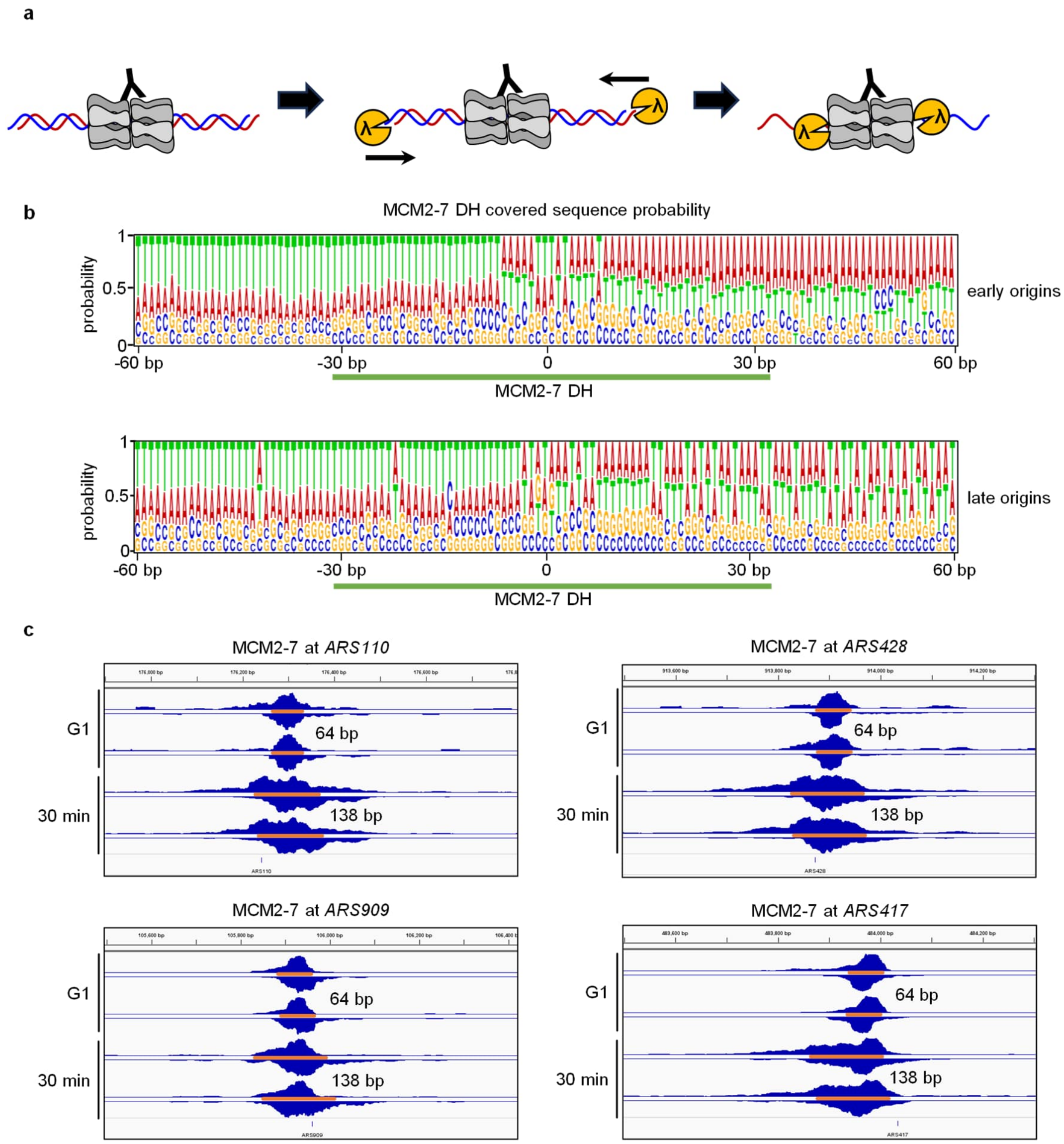

Supplementary Figure 9: ChIP-exo defines the genomic location of the closed McmC2/5 linkage construct.

(**a**) ChIP-exo principle. Crosslinked complexes are immunoprecipitated, first adapters are ligated, before lambda exonuclease (orange) digestion yields stranded footprint DNA with digested 5'-ends, designating the footprint bound to the protein complex with near base pair resolution. (**b**) Nucleotide frequency plots of MCM2-7 DH footprint centred at early (top panel) and late origins (lower panel) in the Mcm2/5 linkage strain. The area covered by MCM2-7 is highlighted (green). (**c**) ChIP-Exo 5.0 single traces of Mcm4 (G1 and 30min released, biol. duplicates) on origins (*ARS110*, *ARS428*, *ARS909*, and *ARS417*). Footprint sizes are indicated and highlighted with orange bars. Traces were visualised by IGViewer (ver. 2.4.14). Source data is provided as a Source Data file.

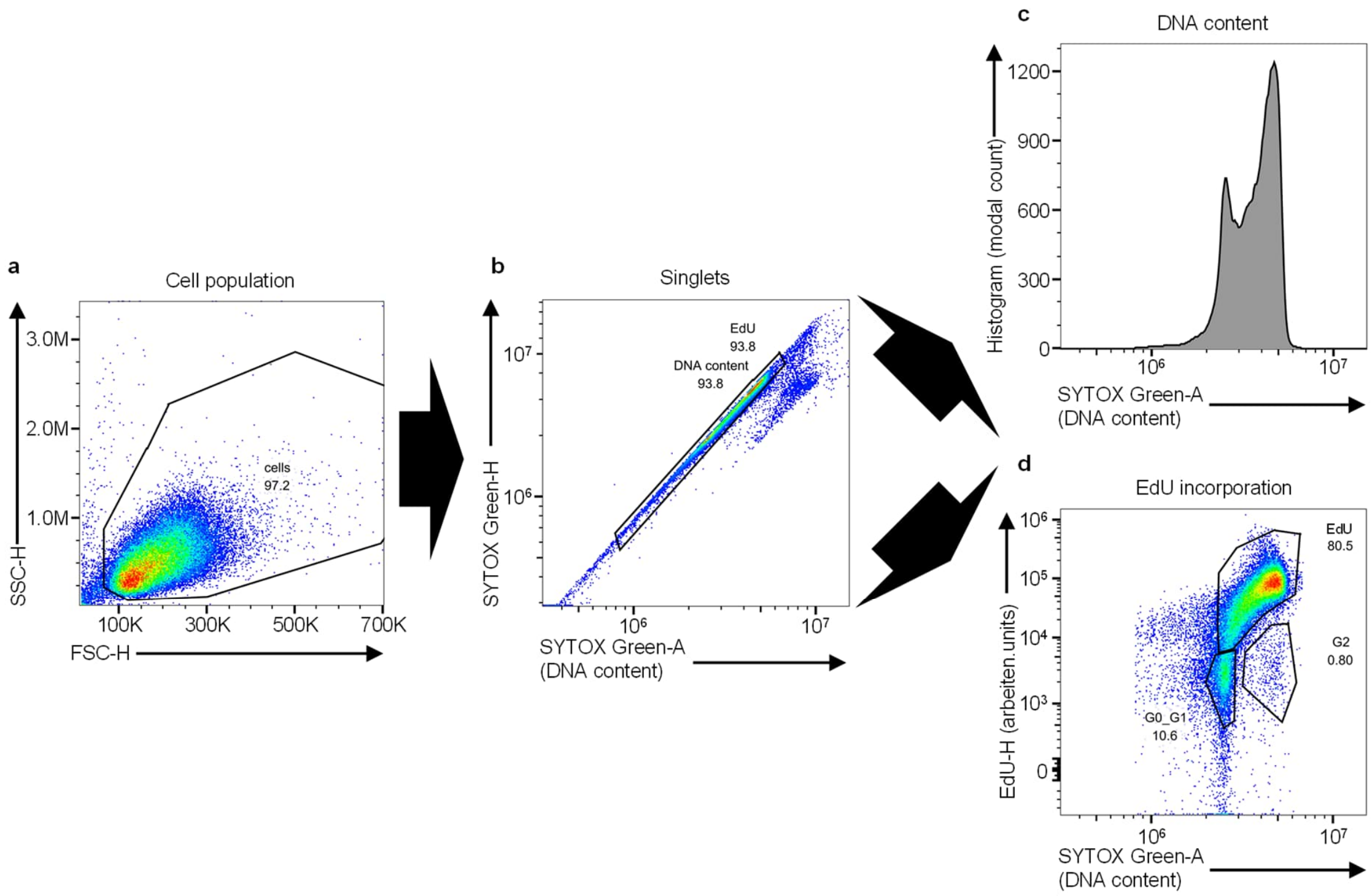

Supplementary Figure 10: Gating strategy for DNA content and EdU incorporation analyses in budding yeast cells.

(**a**) Cells of interest were identified via side scatter height (SSC-H) / forward scatter height (FSC-H) at linear scales (left panel). (**b**) Subsequently, doublets were excluded by SYTOX Green height (SYTOX Green-H) / SYTOX Green area (SYTOX Green-A) at linear scales (middle panel). (**c**) DNA content (SYTOX Green-A) was visualised by a one-dimensional histogram (488 nm laser and a 525/45 nm band pass (BP) filter) on a linear scale (top right panel). (**d**) EdU incorporation as a readout for *de novo* DNA synthesis was recorded using a 637 nm laser and a 667/30 nm BP filter (EdU height, relative fluorescence, and logarithmic scale) for AlexaFluor 647 versus SYTOX Green area (488 nm laser, 525/45 nm filter, and linear scale) (bottom right panel). Population gates are used to measure EdU incorporation at different cell cycle stages. The EdU gate includes both S-phase and G2-phase cells that have incorporated EdU. The numbers indicate the fraction of total analysed cells.

SUPPLEMENTARY TABLES

Supplementary data for yeast strain, oligonucleotides, and MCM2-7 insertion sites used in this study are available as Supplementary data files online.

Supplementary Table 1. Plasmids

Details	Identifier	Source
pKL255 (5xFLAG-hphNT)	pCS762	Labib, K.
pCas9 int-gRNA Ura3	pAR027	Nieduszynski, C.
pInt-ChrVIII hENT1-hsvTK	pAR047	Nieduszynski, C.
pInt-ChrXI hsvTK	pAR048	Nieduszynski, C.
pInt-ChrXV hsvTK	pAR049	Nieduszynski, C.
pFA6a-*MCM2-FRB-221-HisMX6*	pCS642	This study
pFA6a-*MCM2-FRB-712-HisMX6*	pCS756	This study
pFA6a-*MCM3-FRB-37-HisMX6*	pCS1170	This study
pFA6a-*MCM3-FRB-645-HisMX6*	pCS1185	This study
pFA6a-*MCM4-FRB-252-HisMX6*	pCS1171	This study
pFA6a-*MCM4-FRB-739-HisMX6*	pMR72	This study
pFA6a-*MCM5-FKBP-72-kanMX6Δ*	pCS641	This study
pFA6a-*MCM5-FKBP-591-kanMX6Δ*	pCS757	This study
pFA6a-*MCM6-FKBP-124-kanMX6Δ*	pCS1172	This study
pFA6a-*MCM6-FKBP-743-kanMX6Δ*	pMR73	This study
pFA6a-*MCM7-FKBP-30-kanMX6Δ*	pCS1173	This study
pFa6a-*MCM7-FKBP-630-kanMX6Δ*	pMR74	This study
pRS316-*FPR1*	pMR68	This study

All FKBP and FRB proteins represent internal fusions into the Mcm proteins, with the number indicating the amino acid after which the fusion starts.

Supplementary Table 2. Insertion domain and linker sequences for linkage setup

Sequence 5'-3'	Identifier	Source
GCGATCGCAGGTGCTAACACTTGTACTCCTAGAGGTTCAGGTATGTTGCCTTCCGGTATGGCTTCCAGAATCTTGTGGCACGAAATGTGGCACGAAGGTTTGGAAGAAGCCTCTAGATTATACTTCGGTGAAAGAAACGTTAAAGGCATGTTCGAAGTCTTGGAACCATTACATGCTATGATGGAAAGAGGTCCTCAAACTTTGAAGGAAACATCTTTTAATCAAGCTTACGGTAGAGATTTGATGGAAGCACAAGAATGGTGTAGAAAATACATGAAGTCTGGTAACGTAAAAGATTTGTTACAAGCATGGGACTTGTATTACCACGTTTTCAGAAGAATCTCTAAAACTTCTTACCCTTATGATGTTCCTGATTACGCTGGTGCCAATGATGGTGCCGCGATCGCA	FRB with linkers	Samel et al., 2014 and this study
GGCGCGCCAAGTAGTTACCCTTACGACGTTCCAGATTATGCCAGTTTGGGTGGTCCTTCATCCCCTAAAAAGAAAAGAAAAGTCTCCAGAGGTGTTCAAGTTGAAACCATATCTCCAGGTGACGGTAGAACTTTTCCTAAAAGAGGTCAAACTTGTGTTGTCCATTATACAGGCATGTTGGAAGACGGTAAAAAGTTCGATTCTTCAAGAGACAGAAATAAGCCTTTTAAGTTTATGTTGGGTAAACAAGAAGTCATCAGAGGTTGGGAAGAAGGTGTCGCCCAAATGTCCGTAGGTCAACGTGCTAAGTTGACAATTAGTCCAGATTATGCTTACGGTGCAACCGGTCATCCTGGTATTATACCACCTCACGCAACTTTGGTATTCGACGTTGAATTGTTGAAGTTAGAAACATCCTACATCTCCTTCTTAAATTCAGACTTGATAAACAGTAGAACACAAAGAGTAGACGGTCAAATAGGCGCGCC	FKBP with linkers	Samel et al., 2014 and this study

SUPPLEMENTARY REFERENCES

1 Thomas, B. J. & Rothstein, R. Elevated recombination rates in transcriptionally active DNA. *Cell* **56**, 619-630 (1989). https://doi.org/10.1016/0092-8674(89)90584-9

2 Gruber, S. *et al.* Evidence that loading of cohesin onto chromosomes involves opening of its SMC hinge. *Cell* **127**, 523-537 (2006). https://doi.org/10.1016/j.cell.2006.08.048

3 Samel, S. A. *et al.* A unique DNA entry gate serves for regulated loading of the eukaryotic replicative helicase MCM2-7 onto DNA. *Genes Dev* **28**, 1653-1666 (2014). https://doi.org/10.1101/gad.242404.114

4 Reuter, L. M. *et al.* MCM2-7 loading-dependent ORC release ensures genome-wide origin licensing. *Nat Commun* **15**, 7306 (2024). https://doi.org/10.1038/s41467-024-51538-9

5 Rossi, M. J., Lai, W. K. M. & Pugh, B. F. Simplified ChIP-exo assays. *Nat Commun* **9**, 2842 (2018). https://doi.org/10.1038/s41467-018-05265-7
