# Supplementary material for: Mechanisms of MCM2–7 helicase activation and initial DNA melting at near base-pair resolution
Source: Nat Commun. 2026 Jul 23;17:8984. doi: 10.1038/s41467-026-75695-1 (PMC13507265; doi:10.1038/s41467-026-75695-1)
Supplement: Supplementary file 2 [file 41467_2026_75695_MOESM2_ESM.pdf]

Description of Additional Supplementary Files

File Name: Supplementary Data 1

Description: Oligonucleotides used in this study

File Name: Supplementary Data 2

Description: *Saccharomyces cerevisiae* strains used in this study

File Name: Supplementary Data 3

Description: Raw Label-Free Quantification (LFQ) normalised intensity values for ChIP-MS experiments
