# Supplementary material for: Mechanisms of MCM2–7 helicase activation and initial DNA melting at near base-pair resolution
Source: Nat Commun. 2026 Jul 23;17:8984. doi: 10.1038/s41467-026-75695-1 (PMC13507265; doi:10.1038/s41467-026-75695-1)
Supplement: Supplementary file 3 [file 41467_2026_75695_MOESM6_ESM.pdf]

nature portfolio

Corresponding author(s): Christian Speck & L Maximilian Reuter

Last updated by author(s): Jun 25, 2026

# Reporting Summary

Nature Portfolio wishes to improve the reproducibility of the work that we publish. This form provides structure for consistency and transparency in reporting. For further information on Nature Portfolio policies, see our Editorial Policies and the Editorial Policy Checklist.

## Statistics

For all statistical analyses, confirm that the following items are present in the figure legend, table legend, main text, or Methods section.

| n/a | Confirmed | |
|---|---|---|
| ☐ | ☒ | The exact sample size (*n*) for each experimental group/condition, given as a discrete number and unit of measurement |
| ☐ | ☒ | A statement on whether measurements were taken from distinct samples or whether the same sample was measured repeatedly |
| ☐ | ☒ | The statistical test(s) used AND whether they are one- or two-sided<br>*Only common tests should be described solely by name; describe more complex techniques in the Methods section.* |
| ☒ | ☐ | A description of all covariates tested |
| ☐ | ☒ | A description of any assumptions or corrections, such as tests of normality and adjustment for multiple comparisons |
| ☐ | ☒ | A full description of the statistical parameters including central tendency (e.g. means) or other basic estimates (e.g. regression coefficient) AND variation (e.g. standard deviation) or associated estimates of uncertainty (e.g. confidence intervals) |
| ☒ | ☐ | For null hypothesis testing, the test statistic (e.g. *F*, *t*, *r*) with confidence intervals, effect sizes, degrees of freedom and *P* value noted<br>*Give P values as exact values whenever suitable.* |
| ☒ | ☐ | For Bayesian analysis, information on the choice of priors and Markov chain Monte Carlo settings |
| ☒ | ☐ | For hierarchical and complex designs, identification of the appropriate level for tests and full reporting of outcomes |
| ☒ | ☐ | Estimates of effect sizes (e.g. Cohen's *d*, Pearson's *r*), indicating how they were calculated |

*Our web collection on statistics for biologists contains articles on many of the points above.*

## Software and code

Policy information about availability of computer code

Data collection

For Flow cytometry: Samples were measured on an Agilent Novocyte Quanteon using the propriotary system software (NovoExpress 1.6.2) with the following settings: flow rate 14 µL/min, sample volume: 50 µL, mixing at 1,500 rpm every 8 wells for one cycle.
For ChIP-qPCR, data collection was perfomed on a Biorad CF96 qPCR cycler and data was exported from the machine with Biorad CFX Manager (ver. 3.1). Exported data was analysed in MS Excel.
For ChIP-Exo, data collection was performed on an Illumina MiSeq/HiSeq 2500 system. Data was recorded using the propriotary system software.
For PiP-Seq, data collection was performed on an Illumina NextSeq500 system.Data was recorded using the propriotary system software.

Proteomics: Immunoprecipitations (IPs) were processed by single-pot solid-phase-enhanced sample-preparation (SP3) technology [1]. Eluted samples were mixed with reconstitution buffer (50�mM�HEPES pH 8.0, 1% sodium dodecyl sulphate (w/v) SDS, 1% Triton X-100 (v/v), 1% NP-40 (v/v), 1% Tween 20 (v/v), 1% sodium deoxycholate (w/v), 5 mM EDTA pH 8.0, 50 mM NaCl, 1% glycerol (v/v)). Proteins were reduced using 5 mM DTT, followed by alkylation using 20 mM chloroacetamide. Reduced and alkylated protein was transferred to a new tube containing 100% EtOH pre-mixed with Sera-Mag Speedbeads (GE Healthcare, 2 uL of each per sample). Reduced and alkylated protein was added, to a final concentration of 60% EtOH. Protein-bound beads were rinsed three times with 80% EtOH using a magnetic rack, and after the final wash, beads were resuspended in digestion solution (100 mM ammonium bicarbonate pH 8.0) containing 1 ug of Trypsin Gold (Promega). After sonication to disperse the beads, digestions were incubated overnight (18 hrs) at 37°C and dried in a centrifugal evaporator. Total protein digests were dissolved in 0.1% trifluoroacetic acid by shaking (1200rpm) for 30 min and sonication for 10 min, followed by centrifugation (14,000rpm, 5°C and 10 min).
LC-MS/MS analysis was carried out in technical duplicates. Chromatographic separation was performed using an Ultimate 3000 RSLC nano liquid chromatography system (Thermo Scientific) coupled to an Orbitrap Q-Exactive mass spectrometer (Thermo Scientific) via an EASY-Spray source. For LC-MS/MS analysis peptide solutions were injected and loaded onto a trapping column (Acclaim PepMap 100 C18, 100µm × 2cm)

for desalting and concentration at 8 µL/min in 2% acetonitrile, 0.1% trifluoroacetic acid. Peptides were then eluted on-line to an analytical column (EASY-Spray PepMap RSLC C18, 75µm × 75cm). Eluted peptides were analysed by the mass spectrometer operating in positive polarity using a data-dependent acquisition mode. Ions for fragmentation were determined from an initial MS1 survey scan at 70,000 resolution, followed by HCD (Higher-energy Collision Induced Dissociation) of the top 12 most abundant ions at a resolution of 17,500. MS1 and MS2 scan AGC targets were set to 3e6 and 5e4 for maximum injection times of 50 ms and 50 ms respectively. A survey scan m/z range of 400 – 1800 was used, normalised collision energy set to 37% and charge exclusion enabled for unassigned and +1 ions. Dynamic exclusion was set to 30 seconds. References: [1] Hughes, C. S. et al. Ultrasensitive proteome analysis using paramagnetic bead technology. Molecular systems biology 10, 757, doi:10.15252/msb.20145625 (2014).

Data analysis

Flow cytometry: All data were analysed with FlowJo (v10.10.0, BD Bioscience), and an example of the gating strategy is provided (Supplementary Fig. 9).

ChIP-qPCR: CFX Maestro 2.1, MS Excel

Sequencing data:
Primary analysis RTA 2.11.3 and Secondary analysis bcl2fastq2_2.20.0
Sequencing was conducted on an Illumina MiSeq system with 40 base pair single-end reads
Sequencing reads were mapped to sacCer3 genome using Bowtie2 aligner (v. 2.3.5)
Bigwig files were generated for visualizing with genome browser using deeptools (version 3.2.1)
Normalisation of each sample to sequencing read depth using deeptools --normalizeUsing CPM option.
Peaks were called using ChExMix software (v 0.45) .
Further analysis was performed using the following software: FastQC (ver. 0.11.5), bcl2fastq2_2.20.0, ChIPexoQual (https://welch16.github.io/ChIPexoQual/index.html), Bowtie2 aligner (ver. 2.3.5),
deeptools (ver. 3.2.1), bedtools (version 2.27.1), WebLogo 2.8.2 (https://weblogo.berkeley.edu/), MEME Suite (ver. 5.3.0), ChExMix (ver. 0.45),ChIPpeakAnno (ver. 3.38)
ChAsE: Chromatin Analysis and Exploration Tool (https://github.com/hyounesy/ChAsE),
Code for the Ising model computations (https://github.com/XiPacha/Ising-model),
CURVES+ (v3.0 +, http://curvesplus.bsc.es/).

Proteomics data:
Data were processed using the MaxQuant software platform (v1.6.10.43), with database searches carried out by Andromeda search engine against the Swissprot Saccharomyces cerevisiae database (version 20210222, number of entries: 6,049) [2]. A reverse decoy database approach was used at a 1% false discovery rate (FDR) for peptide spectrum matches. Search parameters were as follows: maximum missed cleavages set to 2, fixed modification of cysteine carbamidomethylation and variable modifications of methionine oxidation and protein N-terminal acetylation. Label-free quantification (LFQ) was enabled with an LFQ minimum ratio count of 1. 'Match between runs' function was used with match and alignment time limits of 0.7 and 20 min respectively. For generation of volcano plots, replicates and relative protein abundances were analysed using the Perseus software platform (v1.6.10.43) [3]. Proteins that were not present in at least three out of four replicates in at least one condition were excluded. Missing values for retained proteins were imputed from a normal distribution. Proteins associated with the Replication Initiation GO term were compared with the volcano plot function in Perseus, with an FDR of 0.1 and S0 of 0.1 [4]. References: [2] Cox J, Mann M. MaxQuant enables high peptide identification rates, individualized p.p.b.-range mass accuracies and proteome-wide protein quantification. Nat Biotechnol. 26(12):1367-72, doi: 10.1038/nbt.1511 (2008). [3] Tyanova, S. et al. The Perseus computational platform for comprehensive analysis of (prote)omics data. Nat Methods 13, 731-740, doi:10.1038/nmeth.3901 (2016). [4] Tusher, V. G., Tibshirani, R. & Chu, G. Significance analysis of microarrays applied to the ionizing radiation response. Proc Natl Acad Sci U S A 98, 5116-5121, doi:10.1073/pnas.091062498 (2001).

For manuscripts utilizing custom algorithms or software that are central to the research but not yet described in published literature, software must be made available to editors and reviewers. We strongly encourage code deposition in a community repository (e.g. GitHub). See the Nature Portfolio guidelines for submitting code & software for further information.

# Data

Policy information about availability of data

All manuscripts must include a data availability statement. This statement should provide the following information, where applicable:

- Accession codes, unique identifiers, or web links for publicly available datasets
- A description of any restrictions on data availability
- For clinical datasets or third party data, please ensure that the statement adheres to our policy

Source data are provided with this paper. All raw sequencing data that support the findings of this study have been deposited in the GEO repository with the accession codes GSE173525 and GSE173528. The mass spectrometry proteomics data have been deposited to the ProteomeXchange Consortium via the PRIDE partner repository with the dataset identifier PXD028809.

# Research involving human participants, their data, or biological material

Policy information about studies with human participants or human data. See also policy information about sex, gender (identity/presentation), and sexual orientation and race, ethnicity and racism.

| | |
|---|---|
| Reporting on sex and gender | n/a |
| Reporting on race, ethnicity, or other socially relevant groupings | n/a |
| Population characteristics | n/a |

| | |
|---|---|
| Recruitment | n/a |
| Ethics oversight | n/a |

Note that full information on the approval of the study protocol must also be provided in the manuscript.

# Field-specific reporting

Please select the one below that is the best fit for your research. If you are not sure, read the appropriate sections before making your selection.

☒ Life sciences ☐ Behavioural & social sciences ☐ Ecological, evolutionary & environmental sciences

For a reference copy of the document with all sections, see nature.com/documents/nr-reporting-summary-flat.pdf

# Life sciences study design

All studies must disclose on these points even when the disclosure is negative.

| | |
|---|---|
| Sample size | No statistical methods were used to predetermine sample size. Sample sizes were chosen according to standards in the field: Genomics: 2 biological replicates for adequate power (genome coverage) to detect meaningful differences. Proteomics: 4 biological replicates for sufficient protein coverage and replication. ChIP: A minimal sample size of 3 biological replicates was chosen guided by standards in the field. Flow cytometry. Sample size was chosen by field standards and included a minimum of 40,000 gated events per biological replicate and 3 biological replicates. |
| Data exclusions | No data were excluded |
| Replication | Conclusions in this study, which are based on generated data were verified by at least two individual biological replicates and/or by merging of at least two biologically independent replicates. Minimum biological replicates used: ChIP-qPCR: 3, sequencing: 2, proteomics: 4. All attempts at replication were successful. |
| Randomization | Samples were not randomized, as no allocation into experimental groups was performed. |
| Blinding | The investigators were not blinded during the experimental nor assessment stages due to the intrinsic unbiased nature of the respective assays/analyses and no group allocation was involved in this study. |

# Behavioural & social sciences study design

All studies must disclose on these points even when the disclosure is negative.

| | |
|---|---|
| Study description | *Briefly describe the study type including whether data are quantitative, qualitative, or mixed-methods (e.g. qualitative cross-sectional, quantitative experimental, mixed-methods case study).* |
| Research sample | *State the research sample (e.g. Harvard university undergraduates, villagers in rural India) and provide relevant demographic information (e.g. age, sex) and indicate whether the sample is representative. Provide a rationale for the study sample chosen. For studies involving existing datasets, please describe the dataset and source.* |
| Sampling strategy | *Describe the sampling procedure (e.g. random, snowball, stratified, convenience). Describe the statistical methods that were used to predetermine sample size OR if no sample-size calculation was performed, describe how sample sizes were chosen and provide a rationale for why these sample sizes are sufficient. For qualitative data, please indicate whether data saturation was considered, and what criteria were used to decide that no further sampling was needed.* |
| Data collection | *Provide details about the data collection procedure, including the instruments or devices used to record the data (e.g. pen and paper, computer, eye tracker, video or audio equipment) whether anyone was present besides the participant(s) and the researcher, and whether the researcher was blind to experimental condition and/or the study hypothesis during data collection.* |
| Timing | *Indicate the start and stop dates of data collection. If there is a gap between collection periods, state the dates for each sample cohort.* |
| Data exclusions | *If no data were excluded from the analyses, state so OR if data were excluded, provide the exact number of exclusions and the rationale behind them, indicating whether exclusion criteria were pre-established.* |
| Non-participation | *State how many participants dropped out/declined participation and the reason(s) given OR provide response rate OR state that no participants dropped out/declined participation.* |
| Randomization | *If participants were not allocated into experimental groups, state so OR describe how participants were allocated to groups, and if allocation was not random, describe how covariates were controlled.* |

# Ecological, evolutionary & environmental sciences study design

All studies must disclose on these points even when the disclosure is negative.

| | |
|---|---|
| Study description | *Briefly describe the study. For quantitative data include treatment factors and interactions, design structure (e.g. factorial, nested, hierarchical), nature and number of experimental units and replicates.* |
| Research sample | *Describe the research sample (e.g. a group of tagged Passer domesticus, all Stenocereus thurberi within Organ Pipe Cactus National Monument), and provide a rationale for the sample choice. When relevant, describe the organism taxa, source, sex, age range and any manipulations. State what population the sample is meant to represent when applicable. For studies involving existing datasets, describe the data and its source.* |
| Sampling strategy | *Note the sampling procedure. Describe the statistical methods that were used to predetermine sample size OR if no sample-size calculation was performed, describe how sample sizes were chosen and provide a rationale for why these sample sizes are sufficient.* |
| Data collection | *Describe the data collection procedure, including who recorded the data and how.* |
| Timing and spatial scale | *Indicate the start and stop dates of data collection, noting the frequency and periodicity of sampling and providing a rationale for these choices. If there is a gap between collection periods, state the dates for each sample cohort. Specify the spatial scale from which the data are taken* |
| Data exclusions | *If no data were excluded from the analyses, state so OR if data were excluded, describe the exclusions and the rationale behind them, indicating whether exclusion criteria were pre-established.* |
| Reproducibility | *Describe the measures taken to verify the reproducibility of experimental findings. For each experiment, note whether any attempts to repeat the experiment failed OR state that all attempts to repeat the experiment were successful.* |
| Randomization | *Describe how samples/organisms/participants were allocated into groups. If allocation was not random, describe how covariates were controlled. If this is not relevant to your study, explain why.* |
| Blinding | *Describe the extent of blinding used during data acquisition and analysis. If blinding was not possible, describe why OR explain why blinding was not relevant to your study.* |

Did the study involve field work? ☐ Yes ☐ No

## Field work, collection and transport

| | |
|---|---|
| Field conditions | *Describe the study conditions for field work, providing relevant parameters (e.g. temperature, rainfall).* |
| Location | *State the location of the sampling or experiment, providing relevant parameters (e.g. latitude and longitude, elevation, water depth).* |
| Access & import/export | *Describe the efforts you have made to access habitats and to collect and import/export your samples in a responsible manner and in compliance with local, national and international laws, noting any permits that were obtained (give the name of the issuing authority, the date of issue, and any identifying information).* |
| Disturbance | *Describe any disturbance caused by the study and how it was minimized.* |

# Reporting for specific materials, systems and methods

We require information from authors about some types of materials, experimental systems and methods used in many studies. Here, indicate whether each material, system or method listed is relevant to your study. If you are not sure if a list item applies to your research, read the appropriate section before selecting a response.

### Materials & experimental systems

| n/a | Involved in the study | |
|---|---|---|
| ☐ | ☒ | Antibodies |
| ☐ | ☐ | Eukaryotic cell lines |
| ☐ | ☐ | Palaeontology and archaeology |
| ☐ | ☐ | Animals and other organisms |
| ☐ | ☐ | Clinical data |
| ☐ | ☐ | Dual use research of concern |
| ☐ | ☐ | Plants |

### Methods

| n/a | Involved in the study | |
|---|---|---|
| ☐ | ☒ | ChIP-seq |
| ☐ | ☒ | Flow cytometry |
| ☐ | ☐ | MRI-based neuroimaging |

## Antibodies

| | |
|---|---|
| Antibodies used | anti-FLAG, Sigma, F-1804, 1/300 dilution (various lot #) |
| Validation | The anti-Flag antibody was validated by Western Blotting against FLAG protein and by ChIP-qPCR at on and off target sites of several FLAG-tagged proteins https://www.sigmaaldrich.com/catalog/product/sigma/f1804?lang=de&region=GB |

## Eukaryotic cell lines

Policy information about cell lines and Sex and Gender in Research

| | |
|---|---|
| Cell line source(s) | *State the source of each cell line used and the sex of all primary cell lines and cells derived from human participants or vertebrate models.* |
| Authentication | *Describe the authentication procedures for each cell line used OR declare that none of the cell lines used were authenticated.* |
| Mycoplasma contamination | *Confirm that all cell lines tested negative for mycoplasma contamination OR describe the results of the testing for mycoplasma contamination OR declare that the cell lines were not tested for mycoplasma contamination.* |
| Commonly misidentified lines (See ICLAC register) | *Name any commonly misidentified cell lines used in the study and provide a rationale for their use.* |

## Palaeontology and Archaeology

| | |
|---|---|
| Specimen provenance | *Provide provenance information for specimens and describe permits that were obtained for the work (including the name of the issuing authority, the date of issue, and any identifying information). Permits should encompass collection and, where applicable, export.* |
| Specimen deposition | *Indicate where the specimens have been deposited to permit free access by other researchers.* |
| Dating methods | *If new dates are provided, describe how they were obtained (e.g. collection, storage, sample pretreatment and measurement), where they were obtained (i.e. lab name), the calibration program and the protocol for quality assurance OR state that no new dates are provided.* |

☐ Tick this box to confirm that the raw and calibrated dates are available in the paper or in Supplementary Information.

| | |
|---|---|
| Ethics oversight | *Identify the organization(s) that approved or provided guidance on the study protocol, OR state that no ethical approval or guidance was required and explain why not.* |

Note that full information on the approval of the study protocol must also be provided in the manuscript.

## Animals and other research organisms

Policy information about studies involving animals; ARRIVE guidelines recommended for reporting animal research, and Sex and Gender in Research

| | |
|---|---|
| Laboratory animals | *For laboratory animals, report species, strain and age OR state that the study did not involve laboratory animals.* |
| Wild animals | *Provide details on animals observed in or captured in the field; report species and age where possible. Describe how animals were caught and transported and what happened to captive animals after the study (if killed, explain why and describe method; if released, say where and when) OR state that the study did not involve wild animals.* |
| Reporting on sex | *Indicate if findings apply to only one sex; describe whether sex was considered in study design, methods used for assigning sex. Provide data disaggregated for sex where this information has been collected in the source data as appropriate; provide overall numbers in this Reporting Summary. Please state if this information has not been collected. Report sex-based analyses where performed, justify reasons for lack of sex-based analysis.* |
| Field-collected samples | *For laboratory work with field-collected samples, describe all relevant parameters such as housing, maintenance, temperature, photoperiod and end-of-experiment protocol OR state that the study did not involve samples collected from the field.* |
| Ethics oversight | *Identify the organization(s) that approved or provided guidance on the study protocol, OR state that no ethical approval or guidance was required and explain why not.* |

Note that full information on the approval of the study protocol must also be provided in the manuscript.

# Clinical data

Policy information about clinical studies

All manuscripts should comply with the ICMJE guidelines for publication of clinical research and a completed CONSORT checklist must be included with all submissions.

Clinical trial registration | *Provide the trial registration number from ClinicalTrials.gov or an equivalent agency.*

Study protocol | *Note where the full trial protocol can be accessed OR if not available, explain why.*

Data collection | *Describe the settings and locales of data collection, noting the time periods of recruitment and data collection.*

Outcomes | *Describe how you pre-defined primary and secondary outcome measures and how you assessed these measures.*

# Dual use research of concern

Policy information about dual use research of concern

## Hazards

Could the accidental, deliberate or reckless misuse of agents or technologies generated in the work, or the application of information presented in the manuscript, pose a threat to:

| No | Yes | |
|---|---|---|
| ☒ | ☐ | Public health |
| ☒ | ☐ | National security |
| ☒ | ☐ | Crops and/or livestock |
| ☒ | ☐ | Ecosystems |
| ☒ | ☐ | Any other significant area |

## Experiments of concern

Does the work involve any of these experiments of concern:

| No | Yes | |
|---|---|---|
| ☒ | ☐ | Demonstrate how to render a vaccine ineffective |
| ☒ | ☐ | Confer resistance to therapeutically useful antibiotics or antiviral agents |
| ☒ | ☐ | Enhance the virulence of a pathogen or render a nonpathogen virulent |
| ☒ | ☐ | Increase transmissibility of a pathogen |
| ☒ | ☐ | Alter the host range of a pathogen |
| ☒ | ☐ | Enable evasion of diagnostic/detection modalities |
| ☒ | ☐ | Enable the weaponization of a biological agent or toxin |
| ☒ | ☐ | Any other potentially harmful combination of experiments and agents |

# Plants

Seed stocks | *Report on the source of all seed stocks or other plant material used. If applicable, state the seed stock centre and catalogue number. If plant specimens were collected from the field, describe the collection location, date and sampling procedures.*

Novel plant genotypes | *Describe the methods by which all novel plant genotypes were produced. This includes those generated by transgenic approaches, gene editing, chemical/radiation-based mutagenesis and hybridization. For transgenic lines, describe the transformation method, the number of independent lines analyzed and the generation upon which experiments were performed. For gene-edited lines, describe the editor used, the endogenous sequence targeted for editing, the targeting guide RNA sequence (if applicable) and how the editor was applied.*

Authentication | *Describe any authentication procedures for each seed stock used or novel genotype generated. Describe any experiments used to assess the effect of a mutation and, where applicable, how potential secondary effects (e.g. second site T-DNA insertions, mosiacism, off-target gene editing) were examined.*

# ChIP-seq

## Data deposition

☒ Confirm that both raw and final processed data have been deposited in a public database such as GEO.

☒ Confirm that you have deposited or provided access to graph files (e.g. BED files) for the called peaks.

| | |
|---|---|
| Data access links<br>*May remain private before publication.* | Accession number GSE173525<br><br>https://www.ncbi.nlm.nih.gov/geo/query/acc.cgi?acc=GSE173525<br><br>Mechanisms of MCM2-7 loading and initial DNA melting at near base-pair resolution [Mcm4-Flag ChIP-Exo]<br><br>Accession number GSE173528<br><br>https://www.ncbi.nlm.nih.gov/geo/query/acc.cgi?acc=GSE173528<br>Mechanisms of MCM2-7 loading and initial DNA melting at near base-pair resolution [ssDNA PIP-Seq] |

Files in database submission

| | |
|---|---|
| GSM5269266 | Mcm4-Flag ChIP-Exo G1 arrested, rep1 |
| GSM5269267 | Mcm4-Flag ChIP-Exo G1 arrested, rep2 |
| GSM5269268 | Mcm4-Flag ChIP-Exo G1 arrested, rep3 |
| GSM5269269 | Mcm4-Flag ChIP-Exo 30min released, rep1 |
| GSM5269270 | Mcm4-Flag ChIP-Exo 30min released, rep2 |
| GSM5269271 | Mcm4-Flag ChIP-Exo 30min released, rep3 |
| GSM5269289 | PIP-seq of Mcm4-Flag, G1 arrested,untreated_rep1 |
| GSM5269290 | PIP-seq of Mcm4-Flag, G1 arrested,untreated_rep2 |
| GSM5269291 | PIP-seq of Mcm4-Flag, G1 arrested,treated_rep1 |
| GSM5269292 | PIP-seq of Mcm4-Flag, G1 arrested,treated_rep2 |
| GSM5269293 | PIP-seq of Mcm4-Flag, 15min released,untreated_rep1 |
| GSM5269294 | PIP-seq of Mcm4-Flag, 15min released,untreated_rep2 |
| GSM5269295 | PIP-seq of Mcm4-Flag, 15min released,treated_rep1 |
| GSM5269296 | PIP-seq of Mcm4-Flag, 15min released,treated_rep2 |
| GSM5269297 | PIP-seq of Mcm4-Flag, 22.5min released,untreated_rep1 |
| GSM5269298 | PIP-seq of Mcm4-Flag, 22.5min released,untreated_rep2 |
| GSM5269299 | PIP-seq of Mcm4-Flag, 22.5min released,treated_rep1 |
| GSM5269300 | PIP-seq of Mcm4-Flag, 22.5min released,treated_rep2 |
| GSM5269301 | PIP-seq of Mcm4-Flag, 30min released,untreated_rep1 |
| GSM5269302 | PIP-seq of Mcm4-Flag, 30min released,untreated_rep2 |
| GSM5269303 | PIP-seq of Mcm4-Flag, 30min released,treated_rep1 |
| GSM5269304 | PIP-seq of Mcm4-Flag, 30min released,treated_rep2 |

| | |
|---|---|
| Genome browser session<br>(e.g. UCSC) | n/a |

## Methodology

| | |
|---|---|
| Replicates | All samples for a given ChIP-Exo experiment (biol rep. 2) were analysed by ChexMix individually. The resulting binding events for each individual<br>replicate sample can be found at GEO. --standard binding event reporting mode was used which report events that pass significance threshold in condition as a whole (Binomial, 1.5-fold change, q<0.01). In addition, ChIPexoQual, a Bioconductor package was used to perform ChIP-exo specific quality control. When ChExAlign was used, standard parameters were applied. |
| Sequencing depth | Typical read depth: 3 million single end reads (minimum read length 40). A minimum of 1 million mapped reads were available for the analysis of particular sample. We determined the sequencing depth to be sufficient based on previously reported literature. |
| Antibodies | See antibody section. |
| Peak calling parameters | Read mapping was performed using Bowtie2 (v 2.3.5) using default parameters. ChexMix version 0.45 was run with following custom settings --kldivergencethres -3 or -10 --nomotifs. We also used –exclude option to exclude blacklist regions that includes tRNA genes and the rDNA locus. |
| Data quality | All samples were evaluated for quality using ChIPexoQual, a Bioconductor package designed specifically for ChIP-exo data. The package generates several diagnostic plots and summary measures that enable assessing enrichment and library complexity. Raw sequence quality was also assessed using FastQC, a popular quality control tool for high throughput sequence data. |
| Software | Read mapping was performed using Bowtie2. ChIP-exo specific quality evaluation was performed using ChIPexoQual package. Peak locations were obtained using ChExMix software. Raw sequence quality was also assessed using FastQC, a popular quality control tool for high throughput sequence data. |

## Flow Cytometry

### Plots

Confirm that:

☒ The axis labels state the marker and fluorochrome used (e.g. CD4-FITC).

☒ The axis scales are clearly visible. Include numbers along axes only for bottom left plot of group (a 'group' is an analysis of identical markers).

☒ All plots are contour plots with outliers or pseudocolor plots.

☒ A numerical value for number of cells or percentage (with statistics) is provided.

### Methodology

| | |
|---|---|
| Sample preparation | S. cerevisiae cells were arrested in G1-phase with alpha factor. Upon release into fresh media, samples were withdrawn every 15min for 90min at 25°C and DNA content and EdU incorporation analyses were conducted as described in the M&M section and https://www.degruyterbrill.com/document/doi/10.1515/hsz-2024-0058/html. |
| Instrument | Agilent NovoCyte Flow Cytometer 621220820182 |
| Software | Data was recorded with the Novocyte Quanteon supplied software NovoExpress 1.6.2 and analysed with FlowJo v.10.10.0 |
| Cell population abundance | a minimum of 50,000 single cells were recorded (subjected to EdU analyses) of which a minimum of 40,000 single cells were assessed. |
| Gating strategy | SSC and FSC for cell selection<br>SYTOX Green (488nm 100mW BL;525/45) for DNA staining<br>AlexaFluor 647 (637nm 100mW Red; 667/30) EdU incorporation<br>gating: SSC-H vs FSC-H for cell sizing, Sytox Green-H vs Sytox Green-A for gating of single cells, Count vs Sytox Green-A for DNA content visualisation and Edu-A647-A vs Sytox Green-A for EdU incorporation visualisation (here, population gates for G1, S, and G2 cell cycle stages) were used to evaluate EdU signals and cell cycle stage). An exemplary gating strategy is represented in Supplementary Figure 9. |

☒ Tick this box to confirm that a figure exemplifying the gating strategy is provided in the Supplementary Information.

## Magnetic resonance imaging

### Experimental design

| | |
|---|---|
| Design type | *Indicate task or resting state; event-related or block design.* |
| Design specifications | *Specify the number of blocks, trials or experimental units per session and/or subject, and specify the length of each trial or block (if trials are blocked) and interval between trials.* |
| Behavioral performance measures | *State number and/or type of variables recorded (e.g. correct button press, response time) and what statistics were used to establish that the subjects were performing the task as expected (e.g. mean, range, and/or standard deviation across subjects).* |

### Acquisition

| | |
|---|---|
| Imaging type(s) | *Specify: functional, structural, diffusion, perfusion.* |
| Field strength | *Specify in Tesla* |
| Sequence & imaging parameters | *Specify the pulse sequence type (gradient echo, spin echo, etc.), imaging type (EPI, spiral, etc.), field of view, matrix size, slice thickness, orientation and TE/TR/flip angle.* |
| Area of acquisition | *State whether a whole brain scan was used OR define the area of acquisition, describing how the region was determined.* |

Diffusion MRI ☐ Used ☐ Not used

### Preprocessing

| | |
|---|---|
| Preprocessing software | *Provide detail on software version and revision number and on specific parameters (model/functions, brain extraction, segmentation, smoothing kernel size, etc.).* |
| Normalization | *If data were normalized/standardized, describe the approach(es): specify linear or non-linear and define image types used for transformation OR indicate that data were not normalized and explain rationale for lack of normalization.* |

| | |
|---|---|
| Normalization template | *Describe the template used for normalization/transformation, specifying subject space or group standardized space (e.g. original Talairach, MNI305, ICBM152) OR indicate that the data were not normalized.* |
| Noise and artifact removal | *Describe your procedure(s) for artifact and structured noise removal, specifying motion parameters, tissue signals and physiological signals (heart rate, respiration).* |
| Volume censoring | *Define your software and/or method and criteria for volume censoring, and state the extent of such censoring.* |

## Statistical modeling & inference

| | |
|---|---|
| Model type and settings | *Specify type (mass univariate, multivariate, RSA, predictive, etc.) and describe essential details of the model at the first and second levels (e.g. fixed, random or mixed effects; drift or auto-correlation).* |
| Effect(s) tested | *Define precise effect in terms of the task or stimulus conditions instead of psychological concepts and indicate whether ANOVA or factorial designs were used.* |

Specify type of analysis: ☐ Whole brain ☐ ROI-based ☐ Both

| | |
|---|---|
| Statistic type for inference<br>(See [Eklund et al. 2016]) | *Specify voxel-wise or cluster-wise and report all relevant parameters for cluster-wise methods.* |
| Correction | *Describe the type of correction and how it is obtained for multiple comparisons (e.g. FWE, FDR, permutation or Monte Carlo).* |

## Models & analysis

| n/a | Involved in the study |
|---|---|
| ☐ | ☐ Functional and/or effective connectivity |
| ☐ | ☐ Graph analysis |
| ☐ | ☐ Multivariate modeling or predictive analysis |

| | |
|---|---|
| Functional and/or effective connectivity | *Report the measures of dependence used and the model details (e.g. Pearson correlation, partial correlation, mutual information).* |
| Graph analysis | *Report the dependent variable and connectivity measure, specifying weighted graph or binarized graph, subject- or group-level, and the global and/or node summaries used (e.g. clustering coefficient, efficiency, etc.).* |
| Multivariate modeling and predictive analysis | *Specify independent variables, features extraction and dimension reduction, model, training and evaluation metrics.* |
