# Supplementary material for: Mechanisms of MCM2–7 helicase activation and initial DNA melting at near base-pair resolution
Source: Nat Commun. 2026 Jul 23;17:8984. doi: 10.1038/s41467-026-75695-1 (PMC13507265; doi:10.1038/s41467-026-75695-1)
Supplement: Supplementary file 4 [file 41467_2026_75695_MOESM7_ESM.pdf]

nature portfolio

Peer Review File

Mechanisms of MCM2-7 helicase activation and initial DNA melting at near base-pair resolution

Corresponding Author: Dr L. Maximilian Reuter

This file contains all reviewer reports in order by version, followed by all author rebuttals in order by version.

Version 0:

Reviewer comments:

Reviewer #1

(Remarks to the Author)
The authors have taken a novel approach to explore the mechanism by which the CMG helicase is activated during DNA replication initiation in budding yeast. The motor of the helicase is a hexameric ring of the six MCM2-7 proteins, and two CMG helicases are formed during S-phase from an MCM2-7 double hexamer that was loaded at origins during G1-phase. Firstly, the authors create a 'latch' across the two rings of the double hexamer by fusing Mcm4 to FKBP and Mcm5 to FRB, which proves to be lethal in the presence of rapamycin that bridges FKBP to FRB. When the latch is enforced in G1-phase, the subsequent S-phase is slow and ChIP data indicate that helicase activation likely occurs, but fork progression cannot proceed. Secondly, the authors use the same trick to make linkages between each of the six interfaces in the Mcm2-7 ring, by fusing FKBP-FRB to adjacent subunits, either at the amino termini or the carboxy termini. Interestingly, only the linkages between Mcm2 and Mcm5 are lethal when fused to either amino or carboxy termini. Enforcing the Mcm2-5 linkage (N or C-terminal) in G1-phase inhibits the subsequent S-phase. For the C-terminal linkage, the authors use Mcm4 ChIP-MS to provide evidence that CMG still assembles, despite the replication defect. Collectively, these data suggest that the Mcm2-5 interface might represent the 'gate' through which one strand of DNA is extruded during initiation, as part of the activation process for the CMG helicase. In the remainder of the manuscript, the authors use ChIP-exo to map Mcm2-7 complexes at origins during G1-phase and S-phase, and argue that the Mcm2-7 double hexamer is converted to a larger assembly in presence of the Mcm2-5 linkage, which they interpret as two activated CMG helicases that are unable to pass each other (helicase bypass is an essential step in establishing replication forks at origins during initiation). Overall, the approaches taken are interesting and innovative, and the data indicating that DNA is extruded through the Mcm2-5 gate are an important contribution. At the same time, some of the data are hard to interpret, as discussed below. I think that the manuscript should be a strong candidate for Nature Communications, after a number of issues have been addressed.

Major points:
1. Figure 1 and Figure S5 present ChIP data that are interpreted as reflecting assembly and activation of the CMG helicase. The interpretation is probably correct, but some caution and/or further explanation is required, as recruitment of Mcm2-7 and Cdc45 to origins, as monitored by ChIP, does not necessarily reflect CMG assembly (Mcm2-7 are loaded in G1-phase and Cdc45 is recruited in G1-phase at early origins, together with Sld3, independently of CMG assembly). The defining step in CMG assembly is recruitment of GINS, which is not monitored in these experiments. It's true that the authors monitor Pol epsilon, which should be a proxy for GINS and CMG. Was there some reason why GINS could not be monitored directly? At the very least, the logic needs to explained in the manuscript (i.e. that Pol epsilon likely reflects GINS recruitment and so suggests CMG assembly). Monitoring GINS would be even better.

2. The interpretation of the Mcm4-Mcm5 'latch' experiment in Figure 1 is complicated – the authors argue that the latch will block hexamer rotation, but the data and the subsequent discussion imply that CMG has likely been assembled and activated, leading to unwinding of some origin DNA (the single-strand DNA binding protein RPA is detected at an early origin by ChIP). This all suggests that some rotation of Mcm2-7 has likely occurred despite the latch. Maybe the latch is broken during CMG assembly and activation and then reforms and inhibits the separation of two active CMGs? These possibilities are hard to judge, and the data should probably be discussed more cautiously. Alternatively, the authors might consider whether this experiment, despite being based on an interesting idea, is really something that helps their story (e.g. they could consider starting with the Mcm2-5 gate experiments?)

3. The data in Figures 2-3 indicate that the Mcm2-5 gate is important during initiation. The authors show that enforcing the N-

terminal Mcm2-5 linkage in G1-phase causes a very tight block to replication, whereas the impact of the C-terminal Mcm2-5 linkage is significant but milder. The remainder of the manuscript then focuses on the C-terminal linkage. The reasons for not characterising the molecular defect caused by the N-terminal Mcm2-5 linkage are not clear (it's a 'tighter' phenotype but why is that bad?). It would surely be interesting for the reader to see the impact of enforcing the N-terminal Mcm2-5 linkage in G1-phase for subsequent CMG assembly and activation, using the ChIP-PCR assay (as in Figure 1 and Figure S5), the ChIP-MS assay as in Figure 4 and the ChIP-Exo and PIP-Seq assays (Figures 5-7). In summary, some additional characterisation of the N-terminal Mcm2-5 linkage would be helpful.

4. The ChIP-MS data in Figure 4d indicate that Tof1 is under-represented at nascent forks when cells enter S-phase with the C-terminal Mcm2-5 linkage, compared to control cells. However, the ChIP-MS data in Figure 4b indicate association of Tof1 in presence of the C-terminal Mcm2-5 linkage. How is this discrepancy explained?

5. On page 9, lines 20-26, the authors state "We conclude that the closed Mcm2/5 linkage arrests helicase activation, generating discrete complexes up to 138 bp in size that are immobile, positioned across origin sequences and require 2 times the space of the MCM2-7 DH, exceeding even the average nucleosome-depleted region of 110-130 bp at replication origins. Moreover, DH splitting is not associated with extensive backtracking or sliding of individual CMG complexes; they remain in direct proximity to the MCM2-7 loading site"
How does the idea of helicase activation being arrested fit with the data in Figure 4b, which indicate recruitment of RPA to nascent forks in presence of the closed Mcm2/5 linkage?

6. On page 10, lines 21-25, the authors state "As such, we now understand the GC-content across the yeast replication origins, which is characterised by a sharp "W" pattern right across the MCM2-7 loading site, which is similar between early and late origins (Fig. 6d and e). These sequence properties have the potential to regulate the extrusion of the lagging strand from the MCM2-7 complex during helicase activation."
Similarly, on page 13, lines 13-15 they state "We suggest that the GC maximum at the centre of the DH footprint may regulate DNA unwinding during helicase activation (Fig. 6c-f)."
How does this interpretation of their data fit with earlier data (e.g. Gros et al, 2013, doi: 10.1002/embj.201387278; Gros et al, 2015, Mol Cell, doi: 10.1016/j.molcel.2015.10.022), which indicate that no special DNA sequence features are required for initiation, once Mcm2-7 double hexamers have been loaded?

7. On page 13, lines 4-5, the authors discuss the PIP-Seq data in Figure 7 and state "Surprisingly, initial DNA destabilisation already occurs in G1-phase at the DH interface, likely due to MCM2-7-induced structural changes in the DNA"
Why was such initial DNA destabilisation not detected in previous studies of loaded yeast Mcm2-7 double hexamers by cryoEM?

Minor points:
1. Page numbers would have been helpful!

2. Introduction: Line 20: the authors state that origins "contain specific elements termed A, B1, and B2."
It would be helpful to make clear that the details in the Introduction refer to budding yeast. Also, do all yeast origins have A, B1 and B2 elements?

3. Figure S2C is unclear – how are the timepoints (cyc, G1-90') shown in the figure, as described in the figure legend?

4. Figure 1G: given that Mcm2-7 loading only occurs during G1-phase, why does the Mcm2 signal increase in the presence of the Mcm4-5 'latch' (+ rapamycin), increase upon release from G1-phase?

5. Figure 1I: how does level of unwinding (Rfa1 ChIP) compare in the presence of the Mcm4-5 'latch' (+ rapamycin) with the level that would be seen with 'real' activation of an origin, for example in an experiment in the presence of hydroxyurea?

6. I couldn't find the details for where all the FRB/FKBP domain insertions are made for all combinations of adjacent Mcm2-7 subunits. It would be useful to have a supplementary figure that explains all the details of the insertions (e.g. with alignments that illustrate how the insertions have been made in flexible loops).

7. Figure 3d-g: same comment as for S2C – it is unclear which timepoints were used and how the data in the figure show this. What does (cyc, G1-90') refer to – perhaps this could be rephrased for additional clarity?

8. Figure 4b legend should explain that this experiment was in the presence of rapamycin.

9. The footprinting section on page 9 cites 'ChIP-exo 5.0' without any further explanation or citation of literature, and the non-expert will find this hard to follow, together with the various ChIP-Exo 'footprints' in Figure 5c. The description in the figure legend and Methods section would benefit from a summary of the principles of the technique. The first mention of 'Chip-Exo 5.0' in the main text should include a citation – this is reference 61, which is only cited rather obscurely in the Methods (page 17, line 19), without any direct mention to indicate that this is the original paper on which this manuscript's approach is based.

10. Page 10, lines 1-2: "Thus, the data suggest that the yeast and human MCM2-7 DHs are similar, with DNA-destabilising activity near their centres."
See above – why wasn't destabilisation of DNA seen for the yeast Mcm2-7 DH in cryoEM studies?

Reviewer #2

(Remarks to the Author)
In the present work, Weeks et al. describe a series of studies aimed at better understanding the Mcm2-7 activation and origin melting process in cells. A series of inducible Mcm subunit-fusion constructs were genomically placed into budding yeast cells and used to selective seal off interfaces both within and between Mcm2-7 hexamers upon treatment with rapamycin. The data indicate that an ability to freely open the Mcm2-5 interface and to separate Mcm2-7 double hexamers are particularly important to appropriately timed CMG formation, the recruitment of replication factors, and cell viability. Sequencing approaches highlight where Mcm2-7 is positioned on origins and how the double hexamer footprint expands as DNA is melted.

The paper is well-organized, and appropriate controls have generally been employed for the described experiments. The conclusions appear reasonable based on the data presented, with a few exceptions (noted below), and will be of interest to the field. Some issues should be addressed before a recommendation concerning publication can be rendered.

Principal comments/questions
1. P. 6, lines 10-17. The text states that Mcm10 association is delayed but the timing of its presence matches that of Sld3 and Pol-e. Accordingly, the data do not appear to distinguish between CMG formation, DH splitting and Mcm10 recruitment. In addition, the data could be interpreted to suggest that fusing Mcm4 and Mcm5 interferes with dissociation but not necessarily rotation. Please clarify.
2. Fig. 7 and accompanying text (particularly p. 11, lines 22-24). It is important to test whether locking the Mcm2/5 gate blocks DNA melting vs. unwinding. PIP-seq should be used to examine thymine accessibility for the McmN2/5 and McmC2/5 constructs at different timepoints following rapamycin addition and release of the G1 block to ascertain this.
3. Fig. 1b. Why were only MCM4 and MCM5 chosen for the intra-DH FRB/FKBP fusions? Are the same results achieved with a different pair of subunits?
4. Fig. 2c and elsewhere. Why does closing the N-terminal MCM3/5 interface block cell growth and have certain replication defects? This is attributed to potentially interfering with lagging strand diversion but given this seems hard to envision. Please clarify.
5. Figs 2 and 3. Naively, the logic shown in panel 2b would indicate that there should be no difference closing an MCM interface at either the N- or C-terminal tier. Yet, the data in Fig. 3 show there are clear differences. Please explain this discrepancy and account for why these differences should not be taken as evidence that the system is not just reporting on interface closure, but also on perturbations to subunit interactions that would confound the primary conclusions of the work.
6. Fig. 3b. It appears that the N-terminal MCM2/5 fusions impede replication even in the absence of rapamycin. Please comment on why this is not believed to be problematic for interpreting the data.
7. P. 8. It is stated that linking McmC2/5 at 30 minutes post–G1 release results in de-enrichment or loss of Tof1 (Fig. 4d). However, Supplementary Fig. 6b and Fig. 4c appear show Tof1 enrichment, indicating that Tof1 is associated with the CMG complex. Please clarify.
8. Fig. 4c-d. Have the raw and annotated mass spec data been made available for review? They were not in the downloadable materials.
9. Fig. 6a and legend. Please clearly explain in the text how the stated conclusion is drawn about where local untwisting occurs with respect to the DH interface based on the data shown.
10. Supplementary Figs. 2 and 3. Why do the Mcm2/5 or 5/3 linkages fail to cycle back to 1C and 2C states by 120 min whereas the Mcm4/5 linkage does? What does this behavior imply about their overall function in vivo?
11. Supplementary Figs. 3 and 4. The McmN7/4 and N4/6 constructs display an incomplete progression to 2C by 90 min that is comparable to that seen for McmC2/5. Why is the C2/5 behavior concluded to be significant but not that of N7/4 and N4/6?
Minor points
1. The introduction highlights many proteins that are budding yeast specific (e.g., Sld2, Sld3, etc.). Please note this specificity or also mention the metazoan equivalents. It's also important to note which mechanistic findings are relevant to budding yeast vs. other systems.
2. P. 3, line 7. Bochman and Schwacha (Mol. Cell 2008) were the first to identify the Mcm2/5 gate biochemically. Lyubimov et al. (PNAS 2012) were the first to show structurally that this gate is open in free Mcm2-7 and that this complex forms a left-handed spiral. The two references cited show that this open persists when Cdt1 is present. The sentence and citations should be modified accordingly.
3. P. 3, line 20. Only budding yeast origins contain the A, B1, and B2 elements. Please qualify the statement.
4. Fig. 1a. Please add a description of the three unwinding models.
5. Fig. 1e. Why does the fusion slow EdU incorporation in the absence of rapamycin?
6. Fig. 1e. The maximum fluorescence intensity data for Mcm4/5 latch (45–75 minutes) without rapamycin indicate a replication defect, whereas the cell cycle data in Fig. 1d for the no-rapamycin sample show chromosome doubling during the same time points. This would seem to be an inconsistency, please clarify.
7. P. 5, lines 9–10. FRB is reported to be attached to Mcm4, yet the text refers to “Mcm2-linked FRB–FPR1.” This discrepancy should be corrected.
8. P. 5, line 10. There is a typo: “FFRB” should be corrected to “FRB.”
9. P. 5, line 19. The reference to “Fig. 1d–f” is incorrect and should instead refer to “Fig. 1e and 1f.”
10. On page 5, line 29, the specific recruitment of Mcm2-7 to ARS305, which peaks at 30 minutes post–G1 release (Fig. 1g), is reported. Two possible explanations are proposed; however, neither would seem to clearly account for the observed increase in the Mcm2 signal at 30 minutes post–G1 release. Please clarify further.
11. Fig. 3d-e. The text legend doesn't appear to match the panel legend. McnN2/5 and McmC2/5 are examined in panel d, not just McmN2/5. Similarly, panel e examines McmN5/3 and McmC5/3, not McmC2/5. Please correct.

12. Fig. 3d-g. Please use different shaped symbols for the solid vs. dashed curves to allow the data and fits to be distinguished from each other.
13. Fig. 5e. The legend has insufficient detail to understand/interpret the figure panel.
14. Fig. 6b. Please clarify what an Ising model is and why it was used.
15. Fig. 6f. Either the legend doesn't seem to match the figure panel, or the legend contains insufficient detail to interpret the data shown in the panel. Please clarify.
16. Fig. 7c. The data shown would seem to indicate that unwinding is occurring between the N-terminal interface, not adjacent to it. Please clarify.
17. Methods. Please specify the length and sequence of the linkers used in their various constructs, as well as how the various rapamycin-binding domains were added.

Reviewer #3

(Remarks to the Author)
Summary and overall assessment: This manuscript investigates how the inactive MCM2–7 double hexamer is converted into an active replicative helicase in vivo. Using a rapamycin-inducible FRB–FKBP tethering strategy, the authors restrain specific MCM interfaces and thereby trap normally transient intermediates during helicase activation. Combining these perturbations with cell-cycle analysis, ChIP-based factor recruitment, ChIP-Exo footprinting, and genome-wide detection of initial DNA unwinding, the authors identify a defined activation intermediate and provide functional evidence that mechanical transitions of the double hexamer are coupled to origin DNA melting and replisome assembly.
The study addresses a central problem in chromosome biology: how a licensed origin becomes a moving replication fork in living cells. A major strength of the work is conceptual — the authors use structural knowledge of a large molecular machine to design a conditional in vivo mechanical perturbation. This allows them to trap and analyze a normally inaccessible stage of replication initiation. The conceptual advance of the study lies in trapping and characterizing, in vivo, a discrete helicase activation intermediate that links mechanical remodeling of the MCM double hexamer to origin DNA unwinding and replisome maturation. In doing so, the work bridges structural models of helicase activation with genome-wide measurements in living cells and fills an important gap between origin licensing and fork establishment.
In particular, the systematic interface-tethering analysis is compelling. The observation that perturbation of the Mcm2/5 interface produces a specific helicase-activation phenotype strongly supports the idea that this interface plays a special mechanistic role during activation. Given that the Mcm2/5 interface is the established loading gate of the helicase, the data provide in vivo functional support for the expectation that this gate is reused during strand extrusion in activation.
Overall, this is a technically strong and conceptually interesting study. My suggestions below are aimed at sharpening interpretation and clarifying presentation rather than requesting additional experiments.
Major Comments
1. Distinguishing loading from post-loading activation
In the Results section describing the interface scan (page 7), discussion of helicase loading and strand extrusion during activation is not clearly separated. Prior work from the authors has already established that opening of the Mcm2/5 gate is required for helicase loading. Therefore, lethality associated with Mcm2/5 tethering in a loading context is expected and does not, by itself, address extrusion during activation.
The conceptual advance of the present study lies in separating loading from post-loading activation events. The α-factor arrest and timed rapamycin addition experiments are well designed to distinguish these stages. In particular, separating G1 arrest, G1/S release, and rapamycin addition after S-phase entry allows loading, activation, extrusion, and elongation to be functionally disentangled. Making this distinction more explicit would clarify what is newly demonstrated here relative to prior work.
2. Topological constraint and the nature of the trapped intermediate
The elongation defect observed with the C-terminal Mcm2/5 tether is most parsimoniously explained by failure of CMG bypass rather than by a general defect in helicase activation. If the Mcm2/5 gate remains closed, the two CMGs cannot topologically pass one another; bypass is therefore physically impossible, not merely inefficient.
This topological constraint also informs interpretation of the trapped intermediate. The data are consistent with two activated CMGs that have undergone initial splitting and strand extrusion but remain unable to translocate past one another. Making this logic explicit would sharpen the mechanistic narrative and clarify the structural state being captured.
3. Sld3 release and inter-hexamer linkage
The conclusion that restricting hexamer rotation delays Sld3 release should be tied explicitly to the Mcm4–Mcm5 inter-hexamer latch used in that section. The phrasing currently risks implying that all latches behave equivalently. Clarifying that this conclusion derives specifically from the inter-hexamer Mcm4–Mcm5 crosslink would improve precision.
Relatedly, the manuscript sometimes describes “delayed recruitment” of factors such as Mcm10. The ChIP time courses appear more consistent with prolonged retention or stabilization at origins rather than delayed arrival. Distinguishing recruitment timing from residence time would strengthen the mechanistic interpretation.
4. Structural interpretation of ChIP-Exo footprints
The identification of 64 bp, 72 bp, and ~138 bp footprints is one of the most interesting findings of the paper. However, ChIP-Exo footprint size reflects protection of exonuclease-accessible duplex DNA rather than direct measurement of the physical dimensions of protein complexes. Expansion of the footprint could arise from altered DNA topology, local unwinding, or protein–DNA crosslink geometry.
The data strongly support the existence of an origin-centered unwinding intermediate, but assignment of specific structural states (loaded DH, partially separated hexamers, two CMGs) should be presented as models rather than direct structural readouts.
5. Interpretation of strand separation and elongation
The statement that the Mcm2/5 C-terminal linkage is “consistent with normal strand separation and a block in elongation” is

potentially confusing. If extrusion of the displaced strand is constrained, strand separation cannot be considered fully normal. Clarifying which aspect of unwinding is preserved versus impaired would improve mechanistic precision.
In addition, in Figure 4d Sld3 appears centered around zero, suggesting comparable association at 30 minutes in WT and latched conditions. This should be reconciled with earlier statements regarding delayed Sld3 release.
6. Sequence architecture and unwinding
The GC/AT architecture across origins is intriguing. However, budding yeast origins are intrinsically AT-rich nucleosome-free regions positioned by ORC and MCM loading. The spatial coincidence between unwinding and origin elements may reflect helicase positioning rather than sequence-instructed melting. Presenting this relationship as correlation rather than causation would strengthen the argument.
Minor Comments
1. Please define “DPC bypass” on first use.
2. Clarify explicitly that the first Results section focuses on the inter-hexamer Mcm4–Mcm5 crosslink.
3. In Figure 3, could the different phenotypes observed with N-terminal versus C-terminal linkages reflect topology rather than strict interface specificity? N-terminal tags may bridge hexamers and prevent separation, whereas C-terminal tags may primarily generate intra-hexamer linkages. Briefly addressing this in the Discussion would help interpretation.
4. Clarify the difference between Supplementary Figure 3b/c and main Figure 3b/c (EdU vs DNA content).
5. Clarify the timing of rapamycin addition in the S-phase experiments to avoid confusion about why bulk DNA synthesis is minimally affected.
6. In Figure 4, panels a/b (IP validation) should be discussed separately from panels c/d (biological comparison).
7. Clarify that ARS305 +9 kb represents a distal control region rather than a larger origin fragment.
8. Correct typo in Figure 4 legend (“pre-LC” → “pre-RC”).
9. Add numbered x-axis to Supplementary Figure 7a.
10. Clarify whether heatmap color scales in Figures 5a and 5c are directly comparable.
11. Specify in Figure 5b legend that the data are from G1-phase.
12. Consider combining Figures 5d and 5e for clarity.
13. Avoid repetition of “Interestingly” in successive sentences.
14. Supplementary Figure 6 could include a panel showing WT/C-terminal linkage ratios for easier comparison.
15. Figure 8 schematic: In panel f, the Sld7 symbol appears to remain present in a state where Tof1 is already associated with the N-terminal face of the CMG. Since Sld3/Sld7 are initiation factors expected to dissociate upon/after CMG formation and fork establishment, the cartoon should be corrected for factor presence/absence. More generally, it would help if the schematic explicitly indicated at which step key factors dissociate (e.g., Sld3/Sld7) and when fork protection factors (e.g., Tof1) are gained.

Version 1:

Reviewer comments:

Reviewer #1

(Remarks to the Author)
The authors have addressed all of the points that I raised and I think that the manuscript is now ready for publication in Nature Communications.

Reviewer #2

(Remarks to the Author)
The authors have satisfactory addressed all comments and questions raised in the prior review. No further revisions are suggested.

Reviewer #3

(Remarks to the Author)
The authors have adequately addressed my comments and strengthened the manuscript. I have no further concerns and support publication.

Open Access This Peer Review File is licensed under a Creative Commons Attribution 4.0 International License, which permits use, sharing, adaptation, distribution and reproduction in any medium or format, as long as you give appropriate credit to the original author(s) and the source, provide a link to the Creative Commons license, and indicate if changes were made.
In cases where reviewers are anonymous, credit should be given to 'Anonymous Referee' and the source.
The images or other third party material in this Peer Review File are included in the article's Creative Commons license, unless indicated otherwise in a credit line to the material. If material is not included in the article's Creative Commons license and your intended use is not permitted by statutory regulation or exceeds the permitted use, you will need to obtain permission directly from the copyright holder.
To view a copy of this license, visit https://creativecommons.org/licenses/by/4.0/

Point-by-point response

We would like to thank the reviewers for taking the time to review our manuscript and for providing considered and insightful comments on our work. We have responded to their comments below, and we hope they find the changes satisfactory.

REVIEWER COMMENTS

Reviewer #1 (Remarks to the Author):

The authors have taken a novel approach to explore the mechanism by which the CMG helicase is activated during DNA replication initiation in budding yeast. The motor of the helicase is a hexameric ring of the six MCM2-7 proteins, and two CMG helicases are formed during S-phase from an MCM2-7 double hexamer that was loaded at origins during G1-phase. Firstly, the authors create a 'latch' across the two rings of the double hexamer by fusing Mcm4 to FKBP and Mcm5 to FRB, which proves to be lethal in the presence of rapamycin that bridges FKBP to FRB. When the latch is enforced in G1-phase, the subsequent S-phase is slow and ChIP data indicate that helicase activation likely occurs, but fork progression cannot proceed. Secondly, the authors use the same trick to make linkages between each of the six interfaces in the Mcm2-7 ring, by fusing FKBP-FRB to adjacent subunits, either at the amino termini or the carboxy termini. Interestingly, only the linkages between Mcm2 and Mcm5 are lethal when fused to either amino or carboxy termini. Enforcing the Mcm2-5 linkage (N or C-terminal) in G1-phase inhibits the subsequent S-phase. For the C-terminal linkage, the authors use Mcm4 ChIP-MS to provide evidence that CMG still assembles, despite the replication defect. Collectively, these data suggest that the Mcm2-5 interface might represent the 'gate' through which one strand of DNA is extruded during initiation, as part of the activation process for the CMG helicase. In the remainder of the manuscript, the authors use ChIP-exo to map Mcm2-7 complexes at origins during G1-phase and S-phase, and argue that the Mcm2-7 double hexamer is converted to a larger assembly in presence of the Mcm2-5 linkage, which they interpret as two activated CMG helicases that are unable to pass each other (helicase bypass is an essential step in establishing replication forks at origins during initiation). Overall, the approaches taken are interesting and innovative, and the data indicating that DNA is extruded through the Mcm2-5 gate are an important contribution. At the same time, some of the data are hard to interpret, as discussed below. I think that the manuscript should be a strong candidate for Nature Communications, after a number of issues have been addressed.

Major points:

1. Figure 1 and Figure S5 present ChIP data that are interpreted as reflecting assembly and activation of the CMG helicase. The interpretation is probably correct, but some caution and/or further explanation is required, as recruitment of Mcm2-7 and Cdc45 to origins, as monitored by ChIP, does not necessarily reflect CMG assembly (Mcm2-7 are loaded in G1-phase and Cdc45 is recruited in G1-phase at early origins, together with Sld3, independently of CMG assembly). The defining step in CMG assembly is recruitment of GINS, which is not monitored in these experiments. It's true that the authors monitor Pol epsilon, which should be a proxy for GINS and CMG. Was there some reason why GINS could not be monitored directly? At the very least, the logic needs to explained in the manuscript (i.e. that Pol epsilon likely reflects GINS recruitment and so suggests CMG assembly). Monitoring GINS would be even better.

We appreciate the reviewer's request and have provided the requested data. We observe recruitment of Psf2, an integral component of the GINS complex, indicating that GINS is recruited and therefore CMG assembly progresses normally (Fig. 1). We have updated the manuscript and figure accordingly.

2. The interpretation of the Mcm4-Mcm5 'latch' experiment in Figure 1 is complicated – the authors argue that the latch will block hexamer rotation, but the data and the subsequent discussion imply that CMG has likely been assembled and activated, leading to unwinding of some origin DNA (the single-strand DNA binding protein RPA is detected at an early origin by ChIP). This all suggests that some rotation of Mcm2-7 has likely occurred despite the latch. Maybe the latch is broken during CMG assembly and activation and then reforms and inhibits the separation of two active CMGs? These possibilities are hard to judge, and the data should probably be discussed more cautiously. Alternatively, the authors might consider whether this experiment, despite being based on an interesting idea, is really something that helps their story (e.g. they could consider starting with the Mcm2-5 gate experiments?)

We really appreciate the reviewer's thoughtful comments. The rotation of the two hexamers is estimated to be 60° (PMID: 38760633). Because the FKBP and FRB are connected via a flexible linkage, we agree with the reviewer that the rotation can occur in this context. Thus, strand extrusion is permitted, consistent with our observation of RPA recruitment. That said, DNA synthesis is very slow. We suggest that the two CMGs remain attached to the origin but pull DNA inwards, as observed for SV40 large tumour antigen replication initiation (PMID: 1309914, PMID: 12774115) and the papillomavirus (HPV) E1 replicative helicase (PMID: 12192057), generating loops that, due to their suboptimal organisation, only progress with DNA slowly. Thus, we have revised the manuscript accordingly and provided this model in the discussion. Again, we thank the reviewer for their excellent comment; this revision has strengthened the logic.

3. The data in Figures 2-3 indicate that the Mcm2-5 gate is important during initiation. The authors show that enforcing the N-terminal Mcm2-5 linkage in G1-phase causes a very tight block to replication, whereas the impact of the C-terminal Mcm2-5 linkage is significant but milder. The remainder of the manuscript then focuses on the C-terminal linkage. The reasons for not characterising the molecular defect caused by the N-terminal Mcm2-5 linkage are not clear (it's a 'tighter' phenotype but why is that bad?). It would surely be interesting for the reader to see the impact of enforcing the N-terminal Mcm2-5 linkage in G1-phase for subsequent CMG assembly and activation, using the ChIP-PCR assay (as in Figure 1 and Figure S5), the ChIP-MS assay as in Figure 4 and the ChIP-Exo and PIP-Seq assays (Figures 5-7). In summary, some additional characterisation of the N-terminal Mcm2-5 linkage would be helpful.

We appreciate the reviewer's comment. A tighter phenotype could indeed be good. However, one must also consider at what stage the arrest should occur. As we argued in the context of the C-terminal linkage, we would expect the entire helicase activation reaction to proceed to the point of DNA extrusion and the initiation of DNA synthesis, at which point the extruded ssDNA will be captured by the linkage. However, we did not observe any DNA synthesis at the N-terminal linkage, arguing that something stops at an earlier stage. The MCM2-7 N-terminal domain binds several factors, including Sld3, Cdc45, and GINS. We have now performed cross-linking mass spectrometry to detect which factors associate in the context of the N-terminal linkage. Consistent with our hypothesis, we detected a defect in complex assembly. When comparing the complex composition 30 minutes after release with the G1 timepoint, we identified Sld3, Sld7, and Cdc45 (Supplementary Fig. 5). When comparing the WT and N-term Mcm2/5 linkage

30 minutes after release, it is clear that complex formation is severely impaired in the Mcm2/5 linkage strain, with defects in recruitment of all CDK-dependent helicase activation factors (Supplementary Fig. 5). Thus, the data suggest that the N-terminal linkage interferes with complex assembly. As discussed, the N-terminal domain serves as a site for complex assembly, and the rapamycin-induced linkage appears to restrict access of factors. For these reasons, we did not perform PIP-seq on this construct. We included this information in the supplement and have mentioned it briefly in the manuscript.

In addition, we provide the reviewer with additional information on the N-terminal linkage construct. In the figure below, we show, using ChIP-Exo, that the complex footprint is restricted to 64 bp, consistent with a helicase-activation stopping at an early stage. However, we did not include the data in the manuscript because we did not want to make the overall manuscript too long.

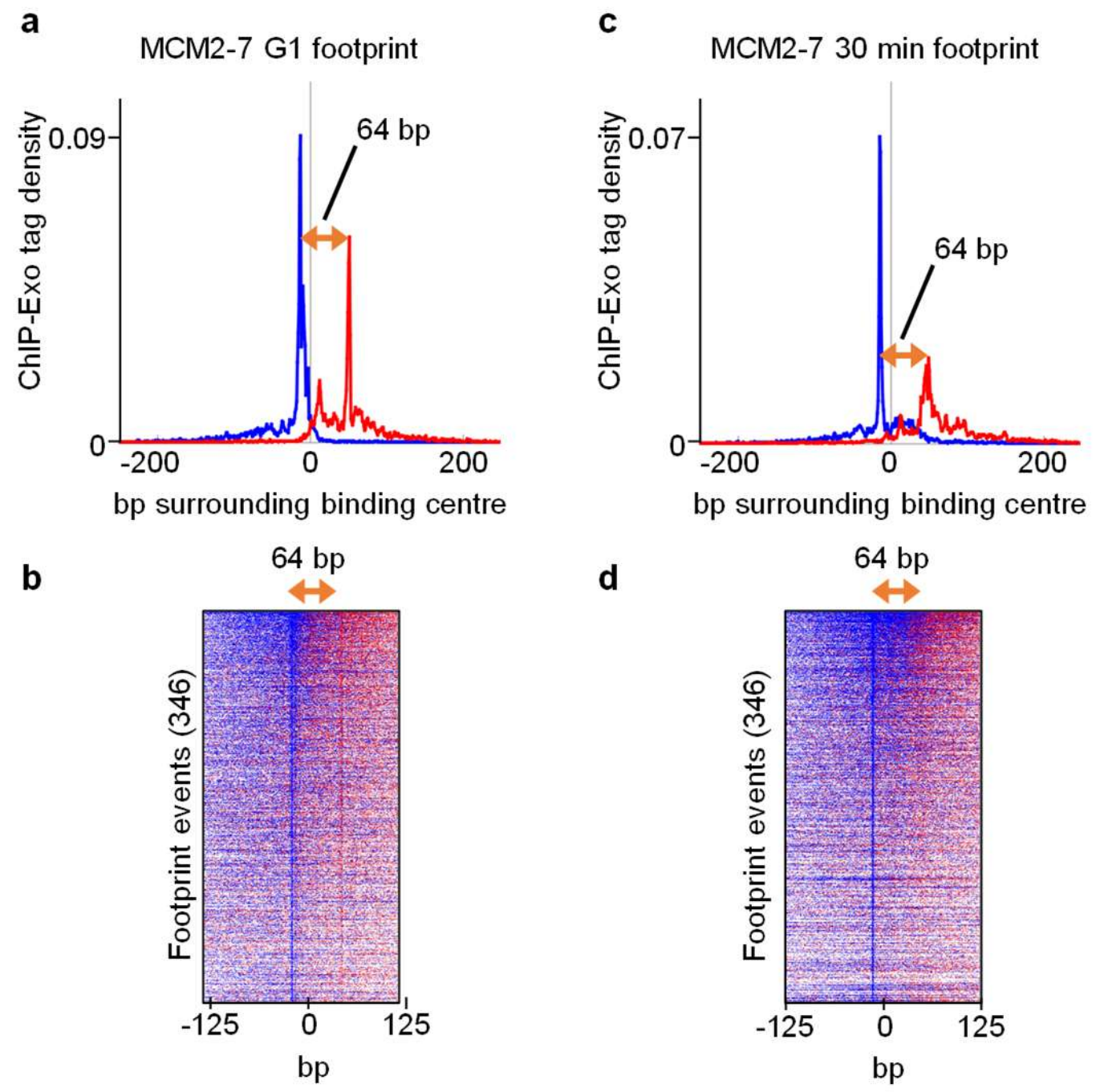

Response figure 1: (a) Fragmented chromatin is subjected to immunoprecipitation, followed by on-bead adapter ligation and exonuclease treatment (PMID: 30030442, PMID: 39181881). Samples are eluted, secondary adapters ligated, and libraries amplified before Illumina sequencing. (a and c) Composite plots of ChIP-Exo 5.0 tag distribution patterns (PMID: 32023130, PMID: 30165373) (forward strand in blue and reverse strand in red) of Mcm4 from G1-arrested (a) and cells released from G1 for 30 minutes (c) treated with rapamycin (100 nM). Distinct footprints of MCM2-7 are displayed based on 346 confirmed core origins ±200 bp. Orange arrows indicate footprint sizes. (b and d) Representative heat maps of ChIP-Exo 5.0 tag enrichments, sorted by decreasing signal, of the MCM2-7 footprints are shown ± 200 bp.

4. The ChIP-MS data in Figure 4d indicate that Tof1 is under-represented at nascent forks when cells enter S-phase with the C-terminal Mcm2-5 linkage, compared to control cells. However, the ChIP-MS data in Figure 4b indicate association of Tof1 in presence of the C-terminal Mcm2-5 linkage. How is this discrepancy explained?

The reason for the difference is that in the context of the WT strain, much more TOF1 becomes recruited (see Supplementary Fig. 6a). Thus, in the C-terminal Mcm2/5 linkage strain, we detect Tof1, but just much less than in the control strain. We now specifically mention this to make the information more accessible.

5. On page 9, lines 20-26, the authors state "We conclude that the closed Mcm2/5 linkage arrests helicase activation, generating discrete complexes up to 138 bp in size that are immobile, positioned across origin sequences and require 2 times the space of the MCM2-7 DH, exceeding even the average nucleosome-depleted region of 110-130 bp at replication origins. Moreover, DH splitting is not associated with extensive backtracking or sliding of individual CMG complexes; they remain in direct proximity to the MCM2-7 loading site". How does the idea of helicase activation being arrested fit with the data in Figure 4b, which indicate recruitment of RPA to nascent forks in presence of the closed Mcm2/5 linkage?

Many thanks for the question. We revised the text to state that replication fork assembly is arrested (absence of Tof1). Many thanks for this really good advice.

6. On page 10, lines 21-25, the authors state "As such, we now understand the GC-content across the yeast replication origins, which is characterised by a sharp "W" pattern right across the MCM2-7 loading site, which is similar between early and late origins (Fig. 6d and e). These sequence properties have the potential to regulate the extrusion of the lagging strand from the MCM2-7 complex during helicase activation." Similarly, on page 13, lines 13-15 they state "We suggest that the GC maximum at the centre of the DH footprint may regulate DNA unwinding during helicase activation (Fig. 6c-f)." How does this interpretation of their data fit with earlier data (e.g. Gros et al, 2013, doi: 10.1002/embj.201387278; Gros et al, 2015, Mol Cell, doi: 10.1016/j.molcel.2015.10.022), which indicate that no special DNA sequence features are required for initiation, once Mcm2-7 double hexamers have been loaded?

Thank you for this excellent question. We suggest that the GC-content may regulate strand separation during helicase activation. So, if the MCM2-7 double-hexamer is pushed to an alternative location, the GC-content can differ, potentially slowing or speeding up strand separation. That said, we have cited the reference and mentioned that an alternative sequence may affect the process of strand separation, dependent on GC-content.

7. On page 13, lines 4-5, the authors discuss the PIP-Seq data in Figure 7 and state "Surprisingly, initial DNA destabilisation already occurs in G1-phase at the DH interface, likely due to MCM2-7-induced structural changes in the DNA". Why was such initial DNA destabilisation not detected in previous studies of loaded yeast Mcm2-7 double hexamers by cryoEM?

Many thanks for this question. We note that potassium permanganate also detects unstacked/twisted bases. Indeed, in the yeast MCM2-7 double-hexamer, base pairs do not follow the standard B-form conformation; hence, they show a signal (PMID: 29078375; PDB:5BK4; Figure 6B). Accordingly, we revised the section in the following way: "Here, we have used PIP-seq to investigate the initial DNA unwinding process in cells (Fig. 7). Potassium permanganate reacts with deformed/twisted DNA or single-stranded thymine bases in the DNA. Interestingly, potassium permanganate reactivity was already detected in G1-phase at the DH interface, likely due to MCM2-7-induced structural changes in the DNA (Fig. 6a and b). Then, during the G1-S

transition, this region expands (Fig. 7c). Concomitantly, we observed an increased dumbbell-shaped footprint (Supplementary Fig. 8c), suggesting DH splitting and DNA unwinding".

Minor points:

1. Page numbers would have been helpful!

We have now included page numbers and line numbering for better accessibility.

2. Introduction: Line 20: the authors state that origins "contain specific elements termed A, B1, and B2." It would be helpful to make clear that the details in the Introduction refer to budding yeast. Also, do all yeast origins have A, B1 and B2 elements?

Many thanks for the question. We have added a citation to the introduction to clarify that we are referring to budding yeast. Indeed, it is thought that all origins contain A-, B1-, and B2-sites, although the B2 sequence had been poorly understood for the longest time (Reuter et al., 2024, PMID: 39181881).

3. Figure S2C is unclear – how are the timepoints (cyc, G1-90') shown in the figure, as described in the figure legend?

Thank you for the comment. To increase clarity, we have added an explanatory figure to Figure S2 to explain how timepoints are displayed on the figure.

4. Figure 1G: given that Mcm2-7 loading only occurs during G1-phase, why does the Mcm2 signal increase in the presence of the Mcm4-5 'latch' (+ rapamycin), increase upon release from G1-phase?

We suggest two reasons for the increased ChIP signal: 1) the latch-mediated arrest renders the CMG complex stationary at the origin, while in the absence of rapamycin, the forks leave the origin quickly, diminishing the ChIP signal at the origin (Fig. 1g, compare ARS305+9kb signals in untreated and rapamycin-treated conditions). 2) CMG-mediated DNA unwinding generates ssDNA-protein contacts, which react more easily with formaldehyde than dsDNA-protein contacts. We have mentioned this in the manuscript.

5. Figure 1I: how does level of unwinding (Rfa1 ChIP) compare in the presence of the Mcm4-5 'latch' (+ rapamycin) with the level that would be seen with 'real' activation of an origin, for example in an experiment in the presence of hydroxyurea?

Thank you very much for this question. We performed the experiment by releasing a WT strain from G1 into hydroxyurea and observed strong RPA recruitment at the replication origin by ChIP-qPCR (Supplementary Fig. 2e), though lower than in the latch context. We note that HU acts much later than the latch-mediated arrest. The latch allows strand extrusion, while HU slows nucleotide incorporation. Thus, these two situations are not completely comparable, as the replication forks can still move slowly in the presence of HU. That said, the level of RPA recruitment we observe with the latch suggests that the two connected CMG helicases can unwind adjacent DNA. We suggest that the two CMGs remain attached to the origin but pull DNA inwards, as observed for SV40 large tumour antigen replication initiation (PMID: 1309914, PMID: 12774115) and the

papillomavirus (HPV) E1 replicative helicase (PMID: 12192057), generating short loops. We have added this explanation to the manuscript.

6. I couldn't find the details for where all the FRB/FKBP domain insertions are made for all combinations of adjacent Mcm2-7 subunits. It would be useful to have a supplementary figure that explains all the details of the insertions (e.g. with alignments that illustrate how the insertions have been made in flexible loops).

Many thanks for the comment. The sites, linkers, and sequences are described in the supplementary material, specifically the section on the integration plasmids and strains. In addition, we added a new supplementary file (Supplementary file 1), that shows alignments, insertion sites, as well as three-dimensional structures of the respective linkages with highlighted insertion sites.

7. Figure 3d-g: same comment as for S2C – it is unclear which timepoints were used and how the data in the figure show this. What does (cyc, G1-90') refer to – perhaps this could be rephrased for additional clarity?

Many thanks. We have addressed this issue in comment 3.

8. Figure 4b legend should explain that this experiment was in the presence of rapamycin.

Thank you, we have updated the text accordingly.

9. The footprinting section on page 9 cites 'ChIP-exo 5.0' without any further explanation or citation of literature, and the non-expert will find this hard to follow, together with the various ChIP-Exo 'footprints' in Figure 5c. The description in the figure legend and Methods section would benefit from a summary of the principles of the technique. The first mention of 'Chip-Exo 5.0' in the main text should include a citation – this is reference 61, which is only cited rather obscurely in the Methods (page 17, line 19), without any direct mention to indicate that this is the original paper on which this manuscript's approach is based.

Many thanks for this really useful comment. We have now added the citation and a very brief explanation in the main manuscript: "This method maps protein-DNA interactions genome-wide at near-single-nucleotide resolution, leveraging λ-exonuclease digestion to identify the boundary of the protein-DNA complex".

10. Page 10, lines 1-2: "Thus, the data suggest that the yeast and human MCM2-7 DHs are similar, with DNA-destabilising activity near their centres." See above – why wasn't destabilisation of DNA seen for the yeast Mcm2-7 DH in cryoEM studies?

We provide a detailed explanation in major point 7.

Reviewer #2 (Remarks to the Author):

In the present work, Weeks et al. describe a series of studies aimed at better understanding the Mcm2-7 activation and origin melting process in cells. A series of inducible Mcm subunit-fusion constructs were genomically placed into budding yeast cells and used to selective seal off interfaces both within and between Mcm2-7 hexamers upon treatment with rapamycin. The data indicate that an ability to freely open the Mcm2-5 interface and to separate Mcm2-7 double hexamers are particularly important to appropriately timed CMG formation, the recruitment of replication factors, and cell viability. Sequencing approaches highlight where Mcm2-7 is positioned on origins and how the double hexamer footprint expands as DNA is melted.

The paper is well-organized, and appropriate controls have generally been employed for the described experiments. The conclusions appear reasonable based on the data presented, with a few exceptions (noted below), and will be of interest to the field. Some issues should be addressed before a recommendation concerning publication can be rendered.

Principal comments/questions

1. P. 6, lines 10-17. The text states that Mcm10 association is delayed but the timing of its presence matches that of Sld3 and Pol-e. Accordingly, the data do not appear to distinguish between CMG formation, DH splitting and Mcm10 recruitment. In addition, the data could be interpreted to suggest that fusing Mcm4 and Mcm5 interferes with dissociation but not necessarily rotation. Please clarify.

Many thanks for the comment. We agree that the slow Mcm10 recruitment is difficult to interpret, but the delayed release is indeed clear-cut. Accordingly, we revised the manuscript. Also, we agree that the 60° rotation during CMG activation can likely occur in the context of the Mcm4/5 construct, due to the linkers used to attach FKBP-FRB. Therefore, we have removed the associated claims regarding the rotation (see also reviewer 1, point 2).

2. Fig. 7 and accompanying text (particularly p. 11, lines 22-24). It is important to test whether locking the Mcm2/5 gate blocks DNA melting vs. unwinding. PIP-seq should be used to examine thymine accessibility for the McmN2/5 and McmC2/5 constructs at different timepoints following rapamycin addition and release of the G1 block to ascertain this.

Many thanks for the comment. We did not observe any DNA synthesis in the context of the N-terminal linkage, arguing that something stops at an earlier stage than the C-terminal gate. The MCM2-7 N-terminal domain binds several factors, including Sld3, Cdc45, and GINS. We have now performed cross-linking mass spectrometry to detect which factors associate in the context of the N-terminal linkage (Supplementary Fig. 5). Consistent with our hypothesis, we detected a defect in complex assembly. When comparing the complex composition 30 minutes after release with the G1 timepoint, we identified Sld3, Sld7, and Cdc45. When comparing the WT and N-term Mcm2/5 linkage 30 minutes after release, it is clear that complex formation is severely impacted in the Mcm2/5 linkage strain (Supplementary Fig. 5). Thus, the data suggest that the N-terminal linkage interferes with complex assembly. As discussed, the N-terminal domain serves as a site for complex assembly, and the rapamycin-induced linkage appears to restrict access of factors. For these reasons, we did not perform PIP-seq on this construct. We updated the manuscript accordingly, briefly mentioning the new proteomic data, to justify why we focused on the C-term construct.

3. Fig. 1b. Why were only MCM4 and MCM5 chosen for the intra-DH FRB/FKBP fusions? Are the same results achieved with a different pair of subunits?

We appreciate the question. We choose Mcm4/5 for the inter-DH latch, as these are the only subunits that are positioned in a favourable orientation (directly opposite each other) and at a distance to create an optimal latch. We have clarified this in the manuscript.

4. Fig. 2c and elsewhere. Why does closing the N-terminal MCM3/5 interface block cell growth and have certain replication defects? This is attributed to potentially interfering with lagging strand diversion but given this seems hard to envision. Please clarify.

Thank you very much for the comment. Accordingly, we have revised our statement: "The Mcm5/3 interface serves as the lagging-strand diversion/exit route (PMID: 32019936) and has also been proposed to function in the handoff of the emerging lagging-strand template to primase (PMID: 37506699). This suggests that the path of single-stranded DNA is dynamic, which rationalises how the restriction of its path by the Mcm3/5 linkage impacts CMG function."

5. Figs 2 and 3. Naively, the logic shown in panel 2b would indicate that there should be no difference closing an MCM interface at either the N- or C-terminal tier. Yet, the data in Fig. 3 show there are clear differences. Please explain this discrepancy and account for why these differences should not be taken as evidence that the system is not just reporting on interface closure, but also on perturbations to subunit interactions that would confound the primary conclusions of the work.

We appreciate the reviewer's comment. We generated multiple constructs to identify one that produces the expected phenotype. Because the N-terminal Mcm2/5 linkage blocked DNA synthesis, we concluded that this construct did not align with our expected results and focused on the C-terminal construct. Based on the reviewer's comment, we have now analysed the underlying defect in the N-terminal linkage. Considering that the linkage is close to a major interaction hub for helicase activation factors, we analysed complex assembly and, unsurprisingly, identified compromised assembly (as the reviewer also expected), with recruitment limited to Sld3 and Cdc45 (Supplementary Figure 5). We included the N-terminal Mcm2/5 data in our original submission in order to be transparent about the potential pitfalls of the N-terminal insertion site. We note that the C-terminal Mcm2/5 linkage impairs Tof1 recruitment, which associates with the N-terminal face of the MCM2-7 complex, distal to the C-terminal linkage. As such, the data are consistent with the C-terminal construct being fully functional, extruding DNA, but interfering with fork passage.

6. Fig. 3b. It appears that the N-terminal MCM2/5 fusions impede replication even in the absence of rapamycin. Please comment on why this is not believed to be problematic for interpreting the data.

The reviewer is right: the Mcm2/5 N-terminal linkage strain displays a small growth defect, as shown in the cell cycle analysis (Fig. 3B). Since the N-terminal Mcm2/5 interface blocked DNA synthesis, we concluded that this construct did not align with our expected results. We included the data to be transparent, but if the reviewer prefers, we can remove it. That said, this kind of weak growth retardation is not unusual in the context of DNA replication mutants.

7. P. 8. It is stated that linking McmC2/5 at 30 minutes post–G1 release results in de-enrichment or loss of Tof1 (Fig. 4d). However, Supplementary Fig. 6b and Fig. 4c appear show Tof1 enrichment, indicating that Tof1 is associated with the CMG complex. Please clarify.

The reason for the difference is that in the context of the WT strain, much more TOF1 becomes recruited (see Supplementary Fig. 6a). Thus, in the C-terminal Mcm2/5 linkage strain, we detect Tof1, but just much less than in the control strain. We mention this now specifically in this section, so the information is more accessible.

8. Fig. 4c-d. Have the raw and annotated mass spec data been made available for review? They were not in the downloadable materials.

Apologies, the data are available in the PRIDE database, with accession codes provided in the data availability statement and the nr-reporting summary file. Also, we provided reviewer access codes. In addition, we have now also included the raw data into the source data file.

9. Fig. 6a and legend. Please clearly explain in the text how the stated conclusion is drawn about where local untwisting occurs with respect to the DH interface based on the data shown.

We have now revised the figure and the text stating "We observed a specific twist in the base pairs near the centre of the protein-DNA complex, which is evident in the structure (Fig. 6, centre) and in the plot of base-pair twist (Fig. 6a, right-hand side), indicating that MCM2-7 alters the DNA structure" and provided a more detailed explanation of the method in the figure legend and of the unwinding location.

10. Supplementary Figs. 2 and 3. Why do the Mcm2/5 or 5/3 linkages fail to cycle back to 1C and 2C states by 120 min whereas the Mcm4/5 linkage does? What does this behavior imply about their overall function in vivo?

The difference reports on the mechanics that break the linkage, which differ between the two constructs. At one time, two motors pull the construct apart (Mcm4/5 linkage), while at another time, the DNA path is modulated, representing different mechanical stress on the system. We have now mentioned, particularly for the latch, two specific models: "We suggest that this defect may represent impaired DNA synthesis, either because the two forks are coupled, resulting in a suboptimal geometry for DNA replication, or because only some MCM2-7 complexes can separate, as the two MCM2-7 motors pull the latch apart. "

11. Supplementary Figs. 3 and 4. The McmN7/4 and N4/6 constructs display an incomplete progression to 2C by 90 min that is comparable to that seen for McmC2/5. Why is the C2/5 behavior concluded to be significant but not that of N7/4 and N4/6?

Please note that comparing cycling cells with cells that undergo arrest and release is never perfect, due to differences in staining. Crucially, the effect is observed even in the absence of rapamycin, suggesting a rapamycin-independent mechanism. As we are interested in the impact of the linkage, we focused on the +/- rapamycin difference.

Minor points

1. The introduction highlights many proteins that are budding yeast-specific (e.g., Sld2, Sld3, etc.). Please note this specificity or also mention the metazoan equivalents. It's also important to note which mechanistic findings are relevant to budding yeast vs. other systems.

Many thanks. We have clarified in the relevant sections and state that this work is carried out in budding yeast. Also, we have added a sentence to the end of the discussion: "Considering that most replication factors are conserved between yeast and human, and that all investigated eukaryotes load MCM2-7 as a double hexamer and form CMG complexes to unwind DNA, it is likely that the mechanistic principles discovered here in budding yeast are conserved in higher eukaryotes, with the exception of DNA sequence specificity".

2. P. 3, line 7. Bochman and Schwacha (Mol. Cell 2008) were the first to identify the Mcm2/5 gate biochemically. Lyubimov et al. (PNAS 2012) were the first to show structurally that this gate is open in free Mcm2-7 and that this complex forms a left-handed spiral. The two references cited show that this open persists when Cdt1 is present. The sentence and citations should be modified accordingly.

Many thanks. We have clarified that MCM2-7 and Cdt1 form a stable complex prior to loading and adopt an open spiral. We have also referred to the publications mentioned here.

3. P. 3, line 20. Only budding yeast origins contain the A, B1, and B2 elements. Please qualify the statement.

We have clarified this as suggested.

4. Fig. 1a. Please add a description of the three unwinding models.

We have added the requested description to the figure legend.

5. Fig. 1e. Why does the fusion slow EdU incorporation in the absence of rapamycin?

We thank the reviewer for the careful observation. Indeed, the discrepancy did not make sense, so we repeated the experiment. We indeed found that there was an issue with the overall click reaction efficiency for these samples that explains the slow EdU signal detection. We have replaced the respective plots for Figure 1e, f, and Supplementary Figure 2d.

6. Fig. 1e. The maximum fluorescence intensity data for Mcm4/5 latch (45–75 minutes) without rapamycin indicate a replication defect, whereas the cell cycle data in Fig. 1d for the no-rapamycin sample show chromosome doubling during the same time points. This would seem to be an inconsistency, please clarify.

Many thanks. We have addressed this issue in the comment above. The data is now consistent with the cell cycle data.

7. P. 5, lines 9–10. FRB is reported to be attached to Mcm4, yet the text refers to "Mcm2-linked FRB–FPR1." This discrepancy should be corrected.

Thank you. This has been corrected as suggested.

8. P. 5, line 10. There is a typo: "FFRB" should be corrected to "FRB."

Thank you. This has been corrected as suggested.

9. P. 5, line 19. The reference to "Fig. 1d–f" is incorrect and should instead refer to "Fig. 1e and 1f."

Thank you. This has been corrected as suggested.

10. On page 5, line 29, the specific recruitment of Mcm2-7 to ARS305, which peaks at 30 minutes post–G1 release (Fig. 1g), is reported. Two possible explanations are proposed; however, neither would seem to clearly account for the observed increase in the Mcm2 signal at 30 minutes post–G1 release. Please clarify further.

We have added a further sentence to clarify this: "Specifically, increased crosslinking through exposed ssDNA with proteins can result in a higher qPCR signal, without an absolute change in protein presence/ recruitment." We note that the depletion of Mcm10 results in a similar effect (PMID: 28818838), which we cited in the manuscript.

11. Fig. 3d-e. The text legend doesn't appear to match the panel legend. McnN2/5 and McmC2/5 are examined in panel d, not just McmN2/5. Similarly, panel e examines McmN5/3 and McmC5/3, not McmC2/5. Please correct.

Thank you. This has been corrected as suggested.

12. Fig. 3d-g. Please use different shaped symbols for the solid vs. dashed curves to allow the data and fits to be distinguished from each other.

Thank you for this useful comment. We have replaced the plots and changed the symbols accordingly. Untreated samples are depicted as a circle (O), and rapamycin-treated samples are represented as a triangle (Δ)

13. Fig. 5e. The legend has insufficient detail to understand/interpret the figure panel.

We have added more detail to the legend to explain that origins, which show an expanded MCM2-7 footprint (Fig. 5c), are mostly early origins. This was achieved by sorting the origins by their Mcm4 ChIP-Exo signal and correlating them with existing replication timing data. This shows that closing the c-terminal Mcm2/5 linkage does not interfere with the inherent replication timing of origins.

14. Fig. 6b. Please clarify what an Ising model is and why it was used.

Thank you. This has been modified as suggested.

15. Fig. 6f. Either the legend doesn't seem to match the figure panel, or the legend contains insufficient detail to interpret the data shown in the panel. Please clarify.

Thank you. The numbering was wrong and has been corrected.

16. Fig. 7c. The data shown would seem to indicate that unwinding is occurring between the N-terminal interface, not adjacent to it. Please clarify.

Thank you. This has been corrected as suggested.

17. Methods. Please specify the length and sequence of the linkers used in their various constructs, as well as how the various rapamycin-binding domains were added.

The length and sequences of the linkers with FRB/FKBP domains have been added as Supplementary Table 4. The insertion sites are detailed in the Supplementary material file, and we have added Supplementary File 1 to show insertion sites and their location on the three-dimensional complex.

Reviewer #3 (Remarks to the Author):

Summary and overall assessment: This manuscript investigates how the inactive MCM2–7 double hexamer is converted into an active replicative helicase in vivo. Using a rapamycin-inducible FRB–FKBP tethering strategy, the authors restrain specific MCM interfaces and thereby trap normally transient intermediates during helicase activation. Combining these perturbations with cell-cycle analysis, ChIP-based factor recruitment, ChIP-Exo footprinting, and genome-wide detection of initial DNA unwinding, the authors identify a defined activation intermediate and provide functional evidence that mechanical transitions of the double hexamer are coupled to origin DNA melting and replisome assembly.
The study addresses a central problem in chromosome biology: how a licensed origin becomes a moving replication fork in living cells. A major strength of the work is conceptual — the authors use structural knowledge of a large molecular machine to design a conditional in vivo mechanical perturbation. This allows them to trap and analyze a normally inaccessible stage of replication initiation. The conceptual advance of the study lies in trapping and characterizing, in vivo, a discrete helicase activation intermediate that links mechanical remodeling of the MCM double hexamer to origin DNA unwinding and replisome maturation. In doing so, the work bridges structural models of helicase activation with genome-wide measurements in living cells and fills an important gap between origin licensing and fork establishment.

In particular, the systematic interface-tethering analysis is compelling. The observation that perturbation of the Mcm2/5 interface produces a specific helicase-activation phenotype strongly supports the idea that this interface plays a special mechanistic role during activation. Given that the Mcm2/5 interface is the established loading gate of the helicase, the data provide in vivo functional support for the expectation that this gate is reused during strand extrusion in activation. Overall, this is a technically strong and conceptually interesting study. My suggestions below are aimed at sharpening interpretation and clarifying presentation rather than requesting additional experiments.

Major Comments

1. Distinguishing loading from post-loading activation

In the Results section describing the interface scan (page 7), discussion of helicase loading and strand extrusion during activation is not clearly separated. Prior work from the authors has already established that opening of the Mcm2/5 gate is required for helicase loading. Therefore, lethality associated with Mcm2/5 tethering in a loading context is expected and does not, by itself, address extrusion during activation.

Many thanks – we clarified that the data suggest a role in helicase loading and/or helicase activation, and clarified that the lethality data cannot distinguish between the two scenarios.

The conceptual advance of the present study lies in separating loading from post-loading activation events. The α-factor arrest and timed rapamycin addition experiments are well designed to distinguish these stages. In particular, separating G1 arrest, G1/S release, and rapamycin addition after S-phase entry allows loading, activation, extrusion, and elongation to be functionally disentangled. Making this distinction more explicit would clarify what is newly demonstrated here relative to prior work.

Many thanks for the comment. We have improved the logic, so it is easier to understand the advance.

2. Topological constraint and the nature of the trapped intermediate
The elongation defect observed with the C-terminal Mcm2/5 tether is most parsimoniously explained by failure of CMG bypass rather than by a general defect in helicase activation. If the Mcm2/5 gate remains closed, the two CMGs cannot topologically pass one another; bypass is therefore physically impossible, not merely inefficient.
This topological constraint also informs interpretation of the trapped intermediate. The data are consistent with two activated CMGs that have undergone initial splitting and strand extrusion but remain unable to translocate past one another. Making this logic explicit would sharpen the mechanistic narrative and clarify the structural state being captured.

Many thanks for the comment. As suggested, we have sharpened the logic and clarified the concept.

We state specifically: ”the elongation defect observed with the C-terminal Mcm2/5 linkage is most likely explained by failure of CMG bypass rather than by a general defect in helicase activation. If the Mcm2/5 gate remains closed, the two CMGs cannot topologically pass one another; bypass is therefore physically impossible, not merely inefficient. As such, the data are consistent with two activated CMGs that have undergone initial splitting and strand extrusion but remain unable to translocate past one another (Fig. 8i)."

3. Sld3 release and inter-hexamer linkage
The conclusion that restricting hexamer rotation delays Sld3 release should be tied explicitly to the Mcm4–Mcm5 inter-hexamer latch used in that section. The phrasing currently risks implying that all latches behave equivalently. Clarifying that this conclusion derives specifically from the inter-hexamer Mcm4–Mcm5 crosslink would improve precision.
Relatedly, the manuscript sometimes describes “delayed recruitment” of factors such as Mcm10. The ChIP time courses appear more consistent with prolonged retention or stabilization at origins rather than delayed arrival. Distinguishing recruitment timing from residence time would strengthen the mechanistic interpretation.

Many thanks for the comment. We agree with the reviewer and have revised the manuscript accordingly, making an explicit reference to the Mcm4/5 latch and focusing on retention. Also, based on the other reviewers' comments, we have removed the rotation statements.

4. Structural interpretation of ChIP-Exo footprints
The identification of 64 bp, 72 bp, and ~138 bp footprints is one of the most interesting findings of the paper. However, ChIP-Exo footprint size reflects protection of exonuclease-accessible duplex DNA rather than direct measurement of the physical dimensions of protein complexes. Expansion of the footprint could arise from altered DNA topology, local unwinding, or protein–DNA crosslink geometry.

We thank the reviewer for this important point. In the context of ORC and the MCM2-7 double-hexamer, we observed footprints of the expected size, but during helicase activation, this could indeed be altered by factors, such as ssDNA or an alternative DNA path. Therefore, we have revised the relevant sections accordingly and mentioned this specifically.

The data strongly support the existence of an origin-centered unwinding intermediate, but assignment of specific structural states (loaded DH, partially separated hexamers, two CMGs) should be presented as models rather than direct structural readouts.

We thank the reviewer for this helpful suggestion. We agree with the reviewer that ChIP-exo can be influenced by several factors, and therefore, we have revised the text and presented the implications as a model.

5. Interpretation of strand separation and elongation
The statement that the Mcm2/5 C-terminal linkage is "consistent with normal strand separation and a block in elongation" is potentially confusing. If extrusion of the displaced strand is constrained, strand separation cannot be considered fully normal. Clarifying which aspect of unwinding is preserved versus impaired would improve mechanistic precision.
In addition, in Figure 4d, Sld3 appears centred around zero, suggesting comparable association at 30 minutes in WT and latched conditions. This should be reconciled with earlier statements regarding the delayed Sld3 release.

Again, many thanks to the reviewer for the excellent comments. We have revised both points as suggested.

6. Sequence architecture and unwinding
The GC/AT architecture across origins is intriguing. However, budding yeast origins are intrinsically AT-rich nucleosome-free regions positioned by ORC and MCM loading. The spatial coincidence between unwinding and origin elements may reflect helicase positioning rather than sequence-instructed melting. Presenting this relationship as correlation rather than causation would strengthen the argument.

Many thanks for the comments. Accordingly, we revised the statements and removed any reference to causation.

Minor Comments
1. Please define "DPC bypass" on first use.

Corrected as suggested.

2. Clarify explicitly that the first Results section focuses on the inter-hexamer Mcm4–Mcm5 crosslink.

Corrected as suggested.

3. In Figure 3, could the different phenotypes observed with N-terminal versus C-terminal linkages reflect topology rather than strict interface specificity? N-terminal tags may bridge hexamers and prevent separation, whereas C-terminal tags may primarily generate intra-hexamer linkages. Briefly addressing this in the Discussion would help interpretation.

Many thanks for the suggestion, but the subunits are too far away to allow trans crosslinking to happen. We explored this further and the N-terminal side appears to block complex assembly, which we now explain in the manuscript.

4. Clarify the difference between Supplementary Figure 3b/c and main Figure 3b/c (EdU vs DNA content).

Many thanks for the comment. Figure 3 b/c shows flow cytometry experiments with cells treated with EdU (2 uM) and +/- rapamycin to investigate both DNA content and EdU incorporation. In contrast, Supplementary Fig. 3b and c only investigate the DNA content under two conditions. Here, the important point was to highlight that closing the 2/5 linkage, either at the N- or C-terminal tier of MCM2-7, has a specific effect on the onset of S-phase, i.e., MCM2-7 DH remodelling and activation (middle panels). Treatment with rapamycin to induce a linkage later (20 min post-release from G1, right panels) did not affect the travelling CMG. Together, this highlights the specific action of the linkage system at the G1-S-phase transition. To improve accessibility of the experiment, we have added a diagram highlighting the treatments and timings (Supplementary Figure 3).

5. Clarify the timing of rapamycin addition in the S-phase experiments to avoid confusion about why bulk DNA synthesis is minimally affected.

A new diagram highlighting the addition of rapamycin was added to Supplementary Fig. 3.

6. In Figure 4, panels a/b (IP validation) should be discussed separately from panels c/d (biological comparison).

Thank you – we have now made this difference clear.

7. Clarify that ARS305 +9 kb represents a distal control region rather than a larger origin fragment.

We have clarified this in the figure legend as suggested.

8. Correct typo in Figure 4 legend (“pre-LC” → “pre-RC”).

We have specified that this is the pre-loading complex (pre-LC).

9. Add numbered x-axis to Supplementary Figure 7a.

A numbered x-axis has been added to the figure.

10. Clarify whether heatmap color scales in Figures 5a and 5c are directly comparable.

Yes, the colour scales are comparable, with blue colour representing the forward strand and red colour describing the reverse strand.

11. Specify in Figure 5b legend that the data are from G1-phase.

We have corrected this.

12. Consider combining Figures 5d and 5e for clarity.

We left this as is because the figure would have three elements without a clear distinction, which would be complicated.

13. Avoid repetition of “Interestingly” in successive sentences.

Corrected, as suggested.

14. Supplementary Figure 6 could include a panel showing WT/C-terminal linkage ratios for easier comparison.

In general, we agree, but comparing different strains is more challenging and error-prone. For this reason, we focused on the volcano plot and enrichment over controls, as this supports confidence in the data.

15. Figure 8 schematic: In panel f, the Sld7 symbol appears to remain present in a state where Tof1 is already associated with the N-terminal face of the CMG. Since Sld3/Sld7 are initiation factors expected to dissociate upon/after CMG formation and fork establishment, the cartoon should be corrected for factor presence/absence. More generally, it would help if the schematic explicitly indicated at which step key factors dissociate (e.g., Sld3/Sld7) and when fork protection factors (e.g., Tof1) are gained.

Many thanks for the comment. We have modified the figure accordingly.
